# Tumor-associated macrophages in meningiomas: a novel biomarker for poor survival outperforming the benefits of T cells

**DOI:** 10.1007/s00401-025-02948-6

**Published:** 2025-10-09

**Authors:** Catharina Lotsch, Rolf Warta, Fang Liu, Gerhard Jungwirth, Carmen Rommel, Mandy Barthel, Katrin Lamszus, Almuth F. Kessler, Niels Grabe, Mario Loehr, Ralf Ketter, Christian Senft, Sybren L. N. Maas, Philipp Sievers, Manfred Westphal, Sandro M. Krieg, Andreas Unterberg, Matthias Simon, Andreas von Deimling, Felix Sahm, David R. Raleigh, Christel Herold-Mende

**Affiliations:** 1 Division of Experimental Neurosurgery, Department of Neurosurgery, Medical Faculty Heidelberg, Heidelberg University, Im Neuenheimer Feld 400, 69120 Heidelberg, Germany; 2 Department of Neurosurgery, University Medical Center Hamburg-Eppendorf, Hamburg, Germany; 3 Department of Neurosurgery, University Hospital Würzburg, Würzburg, Germany; 4 Hamamatsu Tissue Imaging and Analysis Center (TIGA), BIOQUANT, Heidelberg University, Heidelberg, Germany; 5 Department of Neurosurgery, Saarland University, Medical School, Homburg, Germany; 6 Department of Neurosurgery, University of Jena, Jena, Germany; 7 Department of Pathology, Leiden University Medical Center, Leiden, The Netherlands; 8 Department of Pathology, Erasmus MC Cancer Institute, University Medical Center Rotterdam, Rotterdam, The Netherlands; 9 Clinical Cooperation Unit Neuropathology, German Cancer Research Center (DKFZ), Heidelberg, Germany; 10 Department of Neuropathology, Institute of Pathology, Heidelberg University, Heidelberg, Germany; 11 German Cancer Consortium (DKTK), Heidelberg, Germany; 12 Department of Neurosurgery, University Hospital Bonn, Bonn, Germany; 13 Department of Neurosurgery, Bethel Clinic, Bielefeld, Germany; 14 Departments of Radiation Oncology, Neurological Surgery, and Pathology, University of California San Francisco, San Francisco, CA USA

**Keywords:** Meningioma, Macrophages, T cells, Prognosis, DNA methylation, Deconvolution

## Abstract

Tumor-associated macrophages (TAMs) represent the main immune cell population in various brain malignancies, but there is rare knowledge on the functional and, in particular, the prognostic role of TAMs in the meningioma (MGM) microenvironment. Here, we investigated TAM frequencies, activation state, survival-associated changes, and their association with tumor-infiltrating T lymphocytes (TILs) in two independent study samples comprising altogether 680 MGMs. To this end, we performed tissue cytometry analyses, quantified tissue cytokine levels, and integrated previously published TIL infiltration and microarray datasets in the discovery cohort comprising *n* = 195 clinically well-annotated cases. This was complemented by a DNA methylation-based deconvolution approach to predict TAM and TIL infiltration rates using immune cell-specific CpG sites as well as survival associations in an independent validation cohort of *n* = 485 MGMs. Our findings revealed substantial but heterogeneous TAM infiltration in newly diagnosed MGMs, with increased numbers of pro-tumoral TAMs in clinically aggressive tumors. Additional cytokine and transcriptome analyses corroborated the presence of an immunosuppressive niche in TAM-enriched MGMs. Importantly, a high frequency of pro-tumoral TAMs was associated with poor patient outcome, and high TAM infiltration was further identified as an independent prognostic factor for inferior survival, counteracting the beneficial prognostic effect of TILs. Moreover, methylation-based deconvolution analyses confirmed the opposing prognostic roles of TAMs and TILs in the validation cohort. Altogether, higher numbers of TAMs appear to be a hallmark of clinically aggressive behavior in newly diagnosed and recurrent MGMs. Unlike TILs, immunosuppressive TAMs seem to play a dominant role in the immunological landscape of MGMs with a significant negative impact on patient outcome, highlighting pro-tumoral TAMs to be an attractive treatment target in MGMs. Furthermore, our deconvolution approach presents a pipeline to computationally determine TAM and TIL infiltrates in the MGM microenvironment, which might be highly valuable for patient stratification for future immunotherapeutic treatments.

**Supplementary Information:**

The online version contains supplementary material available at 10.1007/s00401-025-02948-6.

## Introduction

Meningiomas (MGMs) are the most common primary intracranial tumors in adults, representing over a third of all the neoplasms in the central nervous system (CNS) [39, 41, 54]. Although the majority of MGMs are slow-growing and histologically benign (WHO grade 1), there is a substantial subset of tumors that exhibit aggressive clinical behavior, resulting in recurrence rates at 5 years of 10–15% for WHO grade 1, 50% for WHO grade 2, and 90% for WHO grade 3 MGMs, respectively [8, 34]. Standard MGM treatment options are limited to surgery and radiotherapy, and despite intensive research efforts, systemic treatment options remain experimental or ineffective [5, 25]. With the breakthrough of immune checkpoint blockade (ICB) therapy in various other tumor types, research on the immunobiology of MGMs has received a great surge of interest, with the efficacy of ICB therapies being under investigation in clinical trials for MGM patients [4, 6, 28, 38]. However, knowledge about the biological impact of the MGM immune microenvironment on disease progression and patient outcome is still incomplete, but could be useful for better selection of patients who may benefit from ICB treatment [22, 57].

In comparison to other malignancies, MGMs are characterized by a low tumor mutational burden, lower lymphocyte numbers, and a predominantly immunosuppressive micromilieu, particularly in higher-grade tumors [57, 59]. Previous studies have shed light on the complex immunological landscape in MGMs and reported also high infiltration of immunosuppressive cells of myeloid origin including myeloid-derived suppressor cells (MDSCs) [27, 44], and tumor-associated macrophages (TAMs) [46, 61]. However, to date, there has been no integrative study analyzing the influence of TAMs on clinical outcomes in a large cohort of MGM patients nor in association with T-cell infiltration.

In tumors, TAMs are recognized as highly heterogeneous and plastic cells with both their phenotype and function being strongly influenced by local microenvironmental cues in the tumor micromilieu [13]. TAMs are still commonly categorized into M1/M2-like macrophage polarization classes, where M1-like TAMs are considered as anti-tumor while M2-like TAMs are presented as pro-tumoral. M2-like TAMs are characterized by increased expression of scavenger receptors (CD163, CD204, and CD206), immune checkpoint ligands (PD-L1), as well as secretion of anti-inflammatory cytokines (IL-10, TGF-β) [13, 23, 32]. In various cancers, these immunosuppressive M2-like TAMs play a key role in tumorigenesis and disease progression and their presence is generally associated with a poor prognosis [9, 13, 35]. Importantly, although these categories may well be oversimplified in the era of single-cell technologies that allow for a much more detailed unraveling of TAM diversity in human cancer, in the present study, we apply this terminology of M1-/M2-like TAMs to describe broader TAM subpopulations, which can be easily identified by routine clinical laboratory techniques (e.g., immunohistological analyses) [29, 33, 35, 48].

In the current study, we assessed TAM frequencies in two large independent cohorts, comprising altogether 680 MGM specimens to elucidate the role of TAMs and pro-tumoral M2-like TAMs in MGMs, and particularly their impact on tumor behavior and patient survival. First, we analyzed TAM infiltration by tissue cytometry in a large and clinically well-annotated discovery cohort (*n* = 195), and found a malignancy- and progression-associated increase of TAM and pro-tumoral M2-like TAM infiltration in MGMs. Notably, in our multivariate analysis, we identified high TAM infiltration as an independent prognostic factor for inferior progression-free survival (PFS) in patients with newly diagnosed MGMs, which counteracted the beneficial impact of higher numbers of tumor-infiltrating T lymphocytes (TILs) in the same study sample. Moreover, in the discovery cohort, we applied a DNA methylation-based deconvolution approach to be able to predict the infiltration rates of TAMs as well as TILs in a second independent tumor cohort (*n* = 485). This computational analysis enabled us to validate our findings on the prognostic roles of both immune cell subsets in MGMs and may further guide the use of TAM- and TIL-associated methylation signatures as novel MGM-specific biomarkers for deconvolving immune cell fractions from DNA methylation data for the next generation of immunotherapy-based clinical trials. Altogether, our data suggest that high TAM infiltration is a hallmark of clinically aggressive MGMs, and further highlight that immunosuppressive pro-tumoral M2-like TAMs represent a promising treatment target for cancer immunotherapy in MGM patients in the future.

## Materials and methods

### Sex as biological variable

Our study examined both male and female patients, and sex was considered as a biological variable and clinically relevant prognostic cofactor in multivariate PFS analyses.

### Discovery cohort tumor specimen collection

The discovery cohort comprised a total of 195 MGM specimens (WHO grade 1 *n* = 43; WHO grade 2 *n* = 97; WHO grade 3 *n* = 62), which were obtained from female and male patients undergoing surgical resection in the Departments of Neurosurgery at University Hospitals Heidelberg, Bonn, Hamburg, Homburg, Frankfurt, and Würzburg, Germany as part of the “FORAMEN” and “KAM” consortia [26, 49, 52]. The use of tissue samples was approved by institutional review boards at each institute in accordance with the Declaration of Helsinki. Written informed consent was obtained from all patients. Tumor specimens were immediately snap-frozen after surgery and stored at − 80 °C until further processing. Tumor cell content ≥ 60% was confirmed for all samples by an experienced neuropathologist (AvD) using hematoxylin & eosin (HE) staining slides of MGM tissues. Tumor specimens with a tumor cell content < 60% and/or with high or extensive necrosis were excluded. Clinical data were collected using a detailed questionnaire and are summarized in Table 1.

**Table 1:** Clinicopathological characteristics of patients with newly diagnosed and recurrent meningioma of the discovery cohort

	Newly diagnosed MGMs (*n* = 120)				Recurrent MGMs (*n* = 75)		
Variable	*n*	Patients (%)	Median (range)		*n*	Patients (%)	Median (range)
Sex							
Male	46	38.33			41	54.66	
Female	74	61.66			34	45.33	
Age at 1st diagnosis (years)			60.8(24.0–87.6)				55.0 (18.0–86.5)
WHO grade							
WHO grade 1	33	27.50			10	13.33	
WHO grade 2	63	52.50			32	42.66	
WHO grade 3	24	20.00			33	44.00	
Subtype							
Transitional	12	10.00			9	12.00	
Fibroblastic	8	6.66					
Meningothelial	8	6.66					
Angiomatous	1	0.83					
Secretory	1	0.83					
Atypical	52	43.33			25	33.33	
Anaplastic	12	10.00			28	37.33	
Rhabdoid	1	0.83			1	1.33	
Papillary	2	1.66					
NA	23	19.16			12	16.00	
Localization							
Convexity	50	41.66			21	28.00	
Cranial base	29	24.16			16	21.33	
Falx	14	11.66			15	20.00	
Parasagittal	17	14.16			13	17.33	
Tentorial	4	3.33			4	5.33	
Other multiple or NA	6	5.00			6	8.00	
Resection grade							
Simpson 1	67	55.83			29	38.66	
Simpson 2	32	26.66			20	26.66	
Simpson 3	16	13.33			12	16.00	
Simpson 4	4	3.33			12	16.00	
Simpson 5	0	0.00			1	1.33	
NA	1	0.83			1	1.33	
Postoperative radiotherapy							
Yes	34	28.33			32	42.66	
No	81	67.50			41	54.66	
NA	5	4.16			2	2.66	
Postoperative chemotherapy							
Yes	0	0.00			7	9.33	
No	117	97.50			64	85.33	
NA	3	2.50			4	5.33	

*n* Number, *NA* Not available, *MGM* Meningioma

### Multicolor immunofluorescence staining

Multicolor immunofluorescence staining was performed on acetone-fixed cryosections (5–7 μm) of the discovery tumor cohort. To quantify TAM and M2-like TAM subpopulations, a combination of primary antibodies specific for CD68 (mouse, Agilent Cat# M0718, RRID:AB_2687454), CD163 (mouse, Bio-Rad Cat# MCA1853T, RRID:AB_2074539), and CD204/MSR1 (rabbit, Sigma-Aldrich Cat# HPA000272, RRID:AB_1846269) were applied as described previously [20]. Briefly, CD163 and CD204 primary antibodies were diluted with Antibody Diluent (Dako). For CD68 staining, primary antibody was coupled to AlexaFluor488 with Zenon labeling kit according to the manufacturer’s instructions (Thermo Fisher Scientific). As secondary antibodies anti-mouse AlexaFluor647 (Thermo Fisher Scientific) and anti-rabbit AlexaFluor555 (Invitrogen) were used for staining of CD163 and CD204, respectively, and diluted with DPBS-containing DAPI (Thermo Fisher Scientific) at 1:1,000 to stain nuclei. Primary anti-CD68 antibody was incubated for 20 min, while other primary and secondary antibodies were incubated for 1 h, Human tonsil tissue and isotype-matched antibodies (rabbit IgG, × 0936, Dako; rat IgG2b, 14–4031, eBioscience; and mouse IgG1, ab91353, Abcam) served as positive and negative controls, respectively.

### Tissue cytometry-based image analysis

Image analysis was performed in a semiautomated set-up at a single-cell level with subsequent phenotypic hierarchical clustering as described before in the discovery cohort [20, 49]. In brief, high-resolution automated multiple image alignments of whole-tissue sections were acquired using a 20 × objective on an Olympus IX51 microscope equipped with a XM10 Camera (Olympus). The Olympus cellSens Dimension Software (version 1.9) was used for image acquisition. Automatic detection and context-based quantification of TAM infiltration by immunofluorescence markers were performed by the StrataQuest Software (version 5.0.1, TissueGnostics GmbH). Regions of interest (ROI) were manually defined depending on histology and quality of the section to exclude adjacent normal brain or necrotic areas. ROIs were drawn in the slide overview using software-based mark-up tools. Quantification was solely performed in areas with high tumor cell content (≥ 60%) and, if present, necrotic areas were generously excluded. Automatically detected cells were visualized in scattergrams and gated according to defined gating schemes for the expression of nucleic and cell surface markers (Supp. Fig. S1). Cutoff between positive- and negative-gated cells was validated by backward gating. To enable robust and reliable cell quantification, strict parameters by means of nuclear size, staining intensity, and background threshold were defined. Cell nuclei were detected based on DAPI staining and used as origin to generate a growing mask over the cytoplasm to the cell membrane. Based on this mask, TAMs were analyzed regarding cell surface expression of CD68 and DAPI (Suppl. Fig. S1). M2-like TAMs were defined by the cell surface expression of CD68 and CD163 and/or CD204. For statistical analysis, the number of cells was given in percent of total cell count (%TCC, defined as total number of DAPI^+^ nuclei without further distinction of cell types). To determine regional variability in large MGM tissue specimens, we additionally analyzed different area sizes in *n* = 3 MGM samples, including ~ 3 mm2 (region of interest = ROI1), ~ 6 mm2 (ROI2), and the whole tumor section (21.78–24.87 mm2; Supp. Fig. S2).

### Luminex assay

Luminex analysis was performed using the Bio-Plex Pro Human Cytokine 27-plex Assay (Bio-Rad) according to manufacturer’s protocol. In short, protein was isolated from a subset of the discovery cohort including 46 MGM specimens (newly diagnosed MGMs: WHO grade 1 *n* = 7, WHO grade 2 *n* = 19, WHO grade 3 *n* = 6; recurrent MGMs: WHO grade 1 *n* = 2, WHO grade 2 *n* = 9, WHO grade 3 *n* = 3) using the Bio-Plex Cell Lysis Kit (Bio-Rad) according to the manufacturer’s instructions. The protein concentrations were determined by Pierce BCA Protein Assay Kit (Thermo Fisher Scientific), and subsequently, all lysates were diluted to 1 mg/mL. In 96-well assay plate, 50 µL of 1 × beads were added and then washed with 100 µL wash buffer twice. Thereafter, 50 µL of standards, samples and controls were added and incubated on a shaker at 850 rpm for 30 min, followed by a washing step. Then, 50 µL 1 × streptavidin-PE were added to each well and the plate was incubated at 850 rpm for 10 min. Thereafter, all wells were washed with 3 × 100 µL. Finally, the beads were resuspended in 125 µL assay buffer, and shaked at 850 rpm for 30 s. Data acquisition and analysis was done using the Luminex 100 Bio-Plex System and the Bio-Plex Manager Software version 6.1 (Bio-Rad). Three analytes had to be excluded from the analysis due to too low protein concentrations (IL-2, IL-5, and IL-15), since protein levels were not detectable in most tissues.

### Microarray analysis

For microarray analysis, we re-used our previously published microarray dataset GSE74385 (*n* = 62 cases) [52] and extended it with additional *n* = 35 MGM cases. As described previously, after removal of adjacent non-tumor tissue or necrotic areas, total RNA was extracted from MGM tissue specimens using the AllPrep DNA/RNA/Protein Kit (Qiagen) according to the manufacturer’s instructions. RNA concentration and integrity were analyzed using the 2100 Bioanalyzer (Agilent). For microarray analysis, 1 µg total RNA of each tumor specimen was subjected to the Genomics Core Facilities of the German Cancer Research Center (DKFZ, Heidelberg, Germany). After quality control, purification, and cDNA synthesis, samples were hybridized to Human HT-12 V.4.0 BeadChip arrays (Illumina) according to the manufacturer’s instructions. Raw-intensity data of the microarrays were further processed and normalized as described before using R programming [www.r-project.org] [52]. The data were analyzed using the following packages: vsn, limma, msigdbr, clusterprofiler, and enrichplot. Following vsn normalization, differential expression was determined by limma using a model that included TAM and TIL infiltration grouping, sex, age at diagnosis, histology, and newly diagnosed or recurrent status. Gene ontology (GO) term and Reactome gene set enrichment analysis (GSEA) were conducted using the Molecular Signatures Database (MSigDB) collections C5 (GO) and C2 (REACTOME). The level of significance for GSEA was set to adjusted *P *value (*P*_adj_) < 0.05.

### DNA methylation-based deconvolution analysis

DNA methylation was used as a surrogate marker to infer immune cell infiltration, based on the principle that different cell types exhibit highly specific DNA methylation signatures that remain stable even in heterogeneous tumor samples. Genomic DNA was isolated from snap-frozen MGM tissue specimens of the Heidelberg discovery cohort using the AllPrep DNA/RNA/Protein Kit (Qiagen) according to the manufacturer’s instructions. DNA methylation was profiled in-house at the Department of Neuropathology (University Hospital Heidelberg) using both the Illumina Infinium HumanMethylation450 (450 K) and Infinium MethylationEPIC (850 K) arrays, which interrogate > 450,000 and > 850,000 CpG sites across the genome, respectively. The independent validation cohort (GSE183647; Choudhury et al. [11]) was profiled exclusively on the EPIC platform. Raw-intensity data (idat files) were processed separately for each platform using the sesame package (openSesame pipeline, default settings), which performs background correction, dye bias equalization, and calculation of β-values (representing the fraction of methylated alleles per CpG locus, ranging from 0 to 1). To ensure comparability between platforms, datasets were merged by common CpG identifiers. CpGs with missing values in any sample were removed, yielding 338,167 high-quality CpGs across 144 tumors. For differential methylation analysis, we applied the limma package, fitting linear models that included quantitative immunofluorescence-based measurements of TAM and TIL densities as continuous outcomes, while adjusting for WHO grade as a covariate to control for confounding [53]. CpGs were then ranked by moderated t-statistics. We chose the t-statistic rather than relying on raw log fold change (logFC) or p values alone, because it reflects both effect size and measurement variability, thereby prioritizing CpGs with robust and reproducible differences. Unlike logFC alone, it penalizes highly variable sites, and unlike p values alone, it is less sensitive to artifacts of large sample size. The empirical Bayes shrinkage of variance estimates implemented in limma further stabilizes results in high-dimensional methylation data. The top 300 hypermethylated and 300 hypomethylated CpGs, were annotated to genes using IlluminaHumanMethylationEPICanno.ilm10b4.hg19 and found to be located throughout the gene (promoter site, gene body, and untranslated regions). To derive biological meaning, GSEA was performed using msigdbr, clusterProfiler, and enrichplot. To construct predictive models of immune infiltration while addressing high dimensionality and multicollinearity, we applied elastic-net regularization (glmnet, *α* = 0.1, 20-fold cross-validation) [17]. Elastic-net regression combines the feature-selection capacity of lasso (L1 penalty) with the stability of ridge regression (L2 penalty), making it particularly well suited for high-dimensional and correlated methylation data. The ridge component stabilizes estimates in the presence of multicollinearity, allowing retention of groups of biologically related CpGs, while the lasso component enforces sparsity by shrinking uninformative coefficients to zero. This balance reduces dimensionality, improves interpretability, and prevents overfitting, thereby increasing generalizability to independent cohorts. The resulting methylation-based predictors compute infiltration scores by linearly combining CpG β values with their respective coefficients (see Supplementary Table S7). Formally, the infiltration score for a given sample *j* is calculated as

$$Scor{e}_{j}={\sum }_{i=1}^{n}{\beta }_{ij}\times {w}_{i},$$

where ${\beta }_{ij}$ is the methylation β value of CpG $i$ in sample $j$, and ${w}_{i}$ is the corresponding coefficient estimated by elastic-net regression.

For the independent validation cohort (GSE183647; Choudhury et al. [11]), raw EPIC array data were processed using the same sesame pipeline and identical preprocessing steps as applied to the Heidelberg cohort. From the resulting β-matrix, we extracted the subset of CpGs selected by our elastic-net model, and infiltration scores were computed using the equation above with coefficients derived from the discovery cohort. Finally, methylation patterns of selected CpGs were visualized using the ComplexHeatmap package, integrating published immune deconvolution signatures from Choudhury et al. [12] to facilitate comparison with independent immune reference datasets.

### Statistical analysis

Data were analyzed by R (Version 4.4.1, survival package) or GraphPad (Version 9.0.0). Differences between two groups (WHO grade 1 vs. grade 2; WHO grade 2 vs. grade 3; WHO grade 1 vs. grade 3; newly diagnosed vs. recurrent MGMs; high vs. low infiltration divided by the median) were calculated using Mann–Whitney U tests for non-parametric data or Student’s unpaired t tests for parametric data. Differences between matched pairs were calculated using Wilcoxon matched-pairs signed-rank test. Data are presented with median values for non-parametric data and with mean values for parametric data. Visualization of correlation heatmap was done by the corrplot package and optimal number of clusters for was determined by calculating the gap statistic using the factoextra package. Comparison between contingency table groups (distribution between molecular groups) were performed by Chi-square tests. Kaplan–Meier plots were used to visualize survival estimates, whereas comparison of survival differences was done by log-rank test and Cox proportional hazard (PH) models. PFS analyses were only performed on patients with Simpson°I-III resection with a follow-up time of at least 60 months, and were otherwise excluded from the survival analysis of the discovery cohort. For recurrence-free survival analysis (LFFR) of the validation cohort, only patients who had undergone gross total resection (GTR) and had a minimum follow-up of 36 months were included. Variables reaching significance in univariate analyses were further included in a multivariate model to assess the independence of clinical covariates. *P* values < 0.05 were considered significant: *, *P* < 0.05; **, *P* < 0.01, ***, *P* < 0.001, ****, *P* < 0.0001).

## Results

### Malignancy- and progression-related infiltration of TAMs and pro-tumoral TAMs in meningioma

**Fig. 1:**
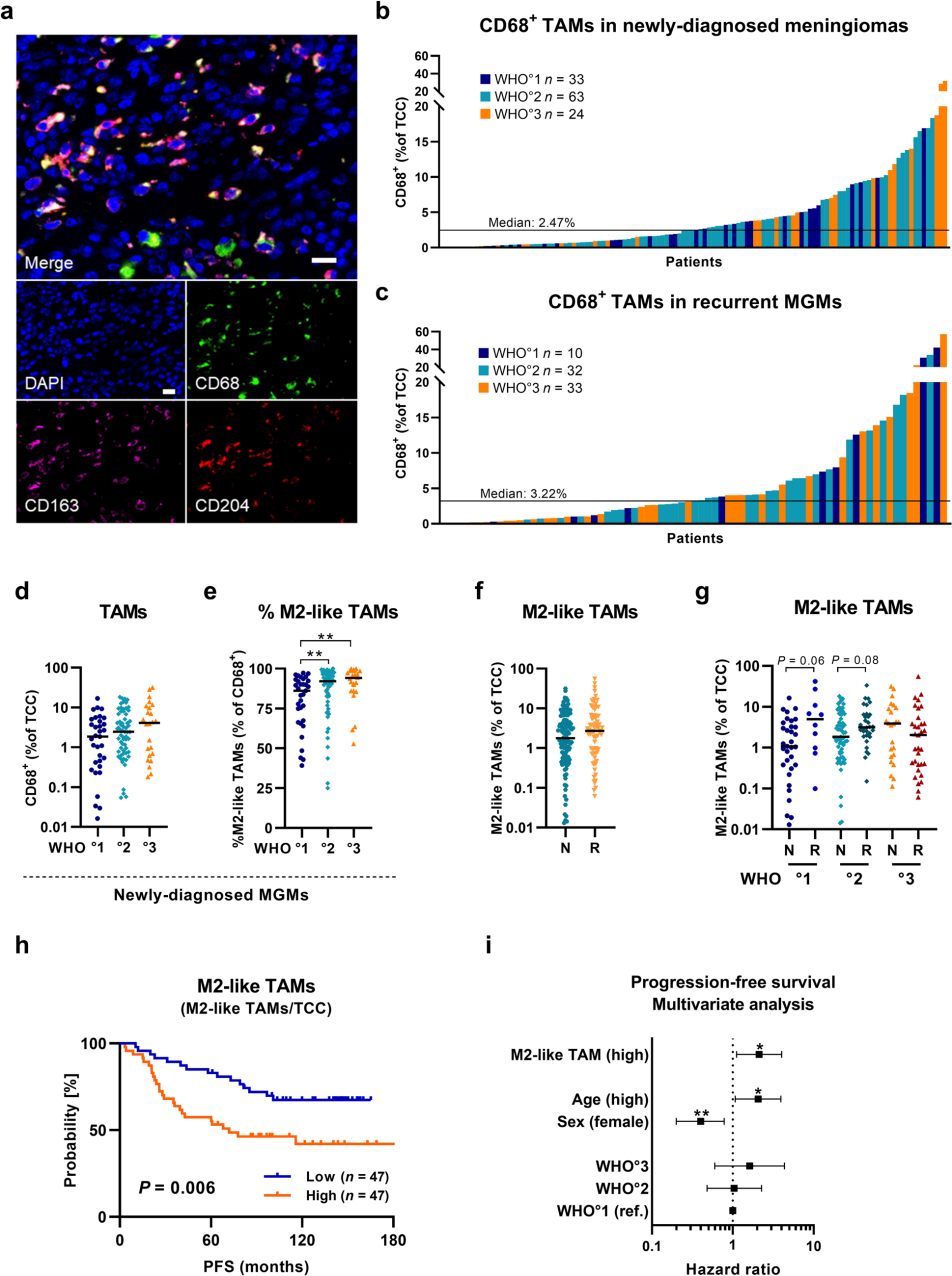
TAM infiltration in newly diagnosed and recurrent meningiomas of the discovery cohort. **a** Representative fluorescent images of TAM staining in MGM tissues. Nuclei stained with DAPI in blue, TAM and M2-like TAM stained with CD68 in green, CD163 in purple, and CD204 in red. Scale bar: 20µm. **b** TAM infiltration (CD68^+^/TCC) in newly diagnosed MGMs. **c** TAM infiltration in recurrent MGMs. **d** TAM infiltration across WHO grades in newly diagnosed MGMs. **e** Proportion of M2-like TAMs (M2-like TAMs/CD68.^+^) across WHO grades in newly diagnosed MGMs. **f** M2-like TAM infiltration (M2-like TAMs/TCC) in newly diagnosed (N) and recurrent (R) MGMs. **g** M2-like TAM infiltration across WHO grades in newly diagnosed (N) and recurrent (R) MGMs. **h** Kaplan–Meier plot for PFS based on high (orange curve) and low (blue curve) M2-like TAM infiltration in newly diagnosed MGMs **i** Multivariate survival analysis for PFS, including prognostic confounders (age, sex, WHO grade) and M2-like TAM infiltration (median split). Statistical significance was calculated using Mann–Whitney U test in (**d-g**), log-rank test in (**h**), and Cox proportional hazard model in (**i**). *MGM* Meningioma, *N* newly diagnosed, *PFS* progression-free survival, *R* recurrent, *ref* Reference, *TAM* tumor-associated macrophage, *TCC* total cell count. Statistical significance: *, *P* < 0.05; **, *P* < 0.01; ***, *P* < 0.001

To study the infiltration of TAMs, we performed multicolor immunofluorescence staining in a discovery cohort, which comprised 195 clinically well-annotated cases including *n* = 120 newly diagnosed and *n* = 75 recurrent MGMs, targeting CD68, a marker that is primarily expressed by macrophages, monocytes, and microglia (Fig. 1a, Table 1). For newly diagnosed MGMs, the study cohort consisted of 33 WHO grade 1, 63 WHO grade 2, and 24 WHO grade 3 tumors, thus containing a high number of clinically aggressive cases. The median age of patients was 60.8 years at the time of first diagnosis with a female-to-male ratio of 1.6 to 1.0. To assess whether tumor recurrence results in altered TAM infiltration, we analyzed 75 recurrent MGMs including a substantial number of high-grade tumors (*n* = 10 WHO grade 1; *n* = 32 WHO grade 2; *n* = 33 WHO grade 3). To increase reliability, we analyzed whole-tissue sections of newly diagnosed (median area: 11.11 mm2) and recurrent MGMs (median area: 10.7 mm2) by tissue cytometry-based image analysis (Suppl. Fig. S1). We quantified TAM infiltration as the number of CD68^+^ TAMs relative to the total cell count (TCC) within tumor sections and observed that TAM infiltration was highly variable across MGM specimens. Additionally, we found small regional variability in TAM infiltration within large MGM tissues when analyzing different sized areas (Suppl. Fig. S2, Suppl. Table S1). Across specimens, TAM percentages varied widely from 0.01 to 31.8% with a median of 2.47% CD68^+^/TCC for newly diagnosed MGMs (Fig. 1b) and shifted toward a higher abundance in recurrent MGMs with a median of 3.22% CD68^+^/TCC (Fig. 1c, Suppl. Fig. S3a). Median TAM infiltration for newly diagnosed MGMs increased with WHO grade: from 1.9% in grade 1 to 2.4% in grade 2 (increase of 26% from grade 1), and to 4.1% in grade 3 tumors (increase of 116% from grade 1), but without reaching a level of significance (Fig. 1d; grade 1 to grade 2, *P* = 0.148; grade 1 to grade 3, *P* = 0.082). Since TAMs can acquire both anti-tumor M1-like or tumor-supportive M2-like phenotypes in the tumor micromilieu, we next examined the polarization status of TAMs by staining for CD204 and CD163, which are two well-known M2-like pro-tumoral TAM markers [13, 23, 32]. Therefore, we characterized CD68^+^ TAMs with an additional CD163^+^ and/or CD204^+^ detection as pro-tumoral and immunosuppressive M2-like TAMs. First, we were interested if the proportion of M2-like TAMs (% of CD68^+^) differed among WHO grades in newly diagnosed MGMs. Here, the median percentage of the M2-like TAM population significantly increased from grade 1 to grade 2, and to WHO grade 3 tumors (Fig. 1e; grade 1 to grade 2, *P* = 0.009; grade 1 to grade 3, *P* = 0.003). Next, we analyzed the infiltration of pro-tumoral M2-like TAMs (M2-like TAMs/TCC) in the whole cohort and found a 1.5-fold higher prevalence of M2-like TAMs in recurrent tumors with a median of 2.72% compared to newly diagnosed MGMs (median of 1.78%; Fig. 1f; *P* = 0.209;). When analyzing M2-like TAM infiltration upon recurrence within the same WHO grade, we observed a threefold increase of pro-tumoral M2-like TAM infiltration in recurrent grade 1 MGMs (Fig. 1g; *P* = 0.055) and a 1.5-fold increase in recurrent grade 2 tumors (Fig. 1g; *P* = 0.078). Similar results were obtained for the proportion of total TAMs when comparing newly diagnosed with recurrent tumors (Suppl. Fig. S3b). Comparison of TAM and pro-tumoral M2-like TAM infiltration in matched pairs of newly diagnosed and recurrent MGMs of *n* = 4 patients likewise demonstrated higher infiltration rates in recurrent tumors (Suppl. Fig. S3c-d). Further, in newly diagnosed MGMs, we found significantly increased levels of TAMs, M2-like TAMs and proportions of M2-like TAMs in prospectively recurring (PR) tumors compared to non-recurring (NR) tumors (minimum follow-up time of 60 months; *P* = 0.001 for TAMs, *P* = 0.001 for M2-like TAMs, and *P* = 0.020 for %M2-like TAMs; Suppl. Fig. S3e-g). Interestingly, prospectively malignant-recurring (PMR) tumors (i.e., a prospective recurrence with increased WHO grading) showed the same trend. In addition, we observed a trend toward higher infiltration of TAMs and higher frequencies of pro-tumoral M2-like TAMs in male patients (*P* = 0.079 and *P* = 0.051; Suppl. Fig. S3h-i), as well as significantly increased numbers of these immune cell subsets in older patients (*P* = 0.032 for TAMs and *P* = 0.016 for %M2-like TAMs; median split at 60.8y; Suppl. Fig. S3j-k) when analyzing newly diagnosed MGMs.

In summary, TAM infiltration in both newly diagnosed and recurrent MGMs was highly heterogeneous yet substantial. We identified significantly higher proportions of pro-tumoral M2-like TAMs in newly diagnosed MGMs of higher-grade tumors, with prospective recurrence and in tumors of older patients (< 60.8 y), as well as increased M2-like TAM frequencies in WHO grade 1 recurrent MGMs and in newly diagnosed tumors of male patients.

### High pro-tumoral M2-like TAM infiltration is an independent prognostic factor for poor progression-free survival in meningiomas

Next, we analyzed whether TAM infiltration has an impact on patient outcome in MGMs. To minimize bias in survival analysis, we included only patients from the discovery cohort who underwent GTR (Simpson°I-III), had no prior treatment, and had a minimum follow-up of 60 months. For the analysis, the patient cohort was then divided into low and high infiltration groups according to the median of TAM and pro-tumoral M2-like TAM infiltration. For newly diagnosed MGMs, survival analysis of the resulting patient cohort (*n* = 94) revealed that high infiltration with pro-tumoral M2-like TAMs was significantly associated with inferior PFS (Fig. 1h; *P* = 0.006). A similar observation for PFS was seen for total TAM infiltration in patients with newly diagnosed MGMs (Suppl. Fig. S3l), whereas in recurrent MGMs, the presence of TAMs had no further impact on PFS after recurrence (Suppl. Fig. S3m).

Subsequently, a multivariate analysis was conducted, incorporating age, sex, and WHO grade as relevant prognostic factors. Importantly, this analysis revealed that high pro-tumoral M2-like TAM infiltration (by median split) is an independent prognostic factor for poor PFS in patients with newly diagnosed MGMs (Fig. 1i, Suppl. Table S2; hazard ratio (HR) = 2.11; *P* = 0.023). In addition, this trend was found to be even more pronounced when analyzing pro-tumoral M2-like TAM infiltration as a Z-transformed continuous variable (M2-like TAM total; Suppl. Fig. S3n; HR = 1.57; *P* = 0.002). Altogether, high numbers of pro-tumoral M2-like TAMs were found to be associated with a poor PFS in patients with newly diagnosed MGMs independent of other prognostic factors.

### Higher TAM infiltration is associated with an immunosuppressive micromilieu in meningiomas

**Fig. 2:**
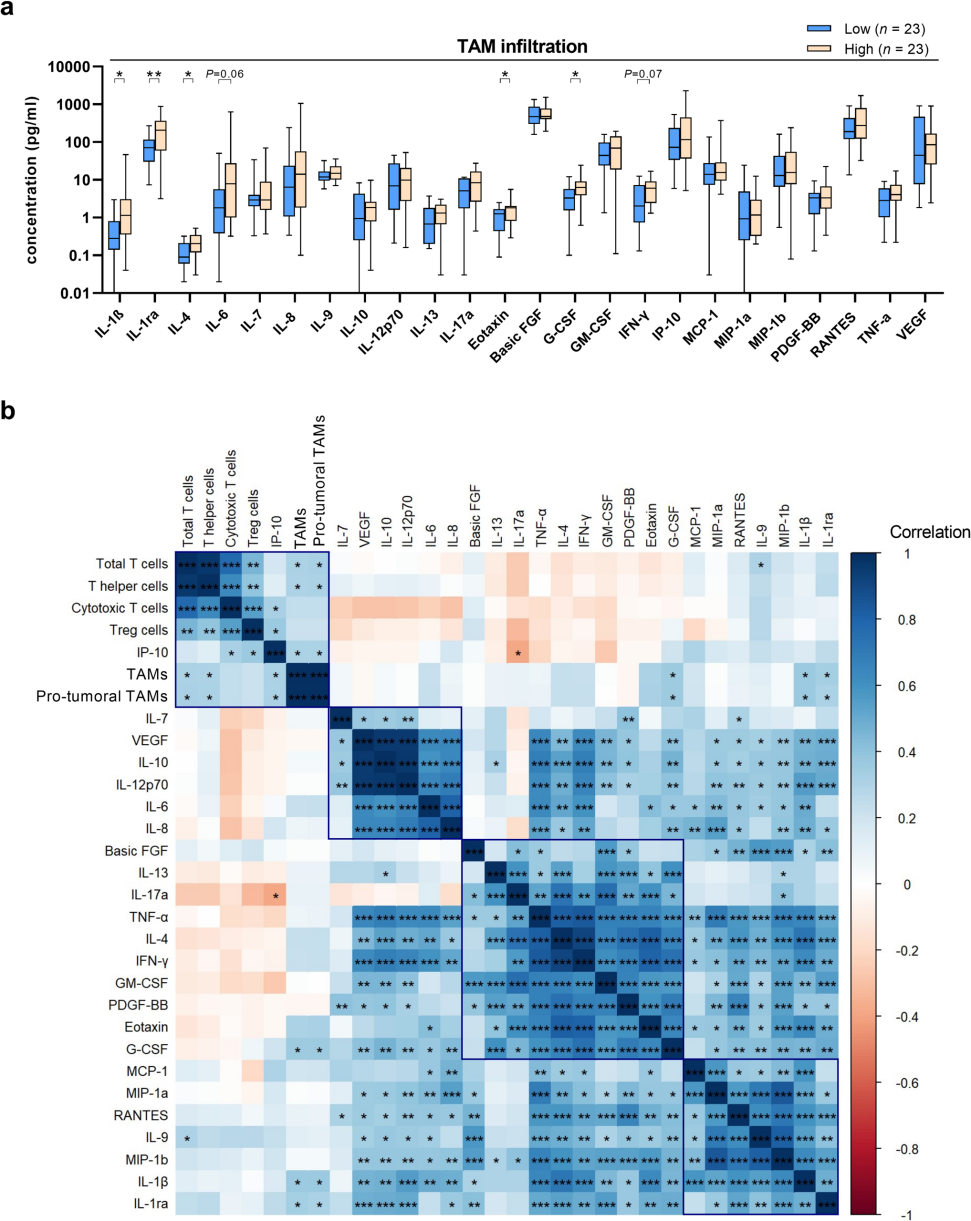
The cyto- and chemokine profile of meningiomas. **a** Concentrations of 24 cytokines and chemokines in MGM tissues (*n* = 46, subset of discovery cohort) assessed by Luminex analysis comparing TAM low (light blue) and TAM high (orange) infiltration in tumor specimens (median split). **b** Correlation matrix of protein concentrations, and TAM and TIL infiltration numbers ordered by Spearman correlation. Statistical significance was calculated in **a** using Mann–Whitney U tests (individually for each analyte) and in **b** using Spearman correlation. *MGM* Meningioma, *PFS* progression-free survival, *TAM* tumor-associated macrophage, *TIL* tumor-infiltrating T lymphocyte. Statistical significance: *, *P* < 0.05; **, *P* < 0.01; ***, *P* < 0.001

TAMs play vital roles in the local tumor milieu by secreting various soluble factors, including chemokines, cytokines, and growth factors, which can in particular influence the attraction and function of effector T cells [13]. To explore the TAM-related cytokine and chemokine milieu in MGM, we performed Luminex analyses of 24 different immune-related cytokines, chemokines, and growth factors in a subset of 46 tissue samples (WHO grade 1 *n* = 9, WHO grade 2 *n* = 28, WHO grade 3 *n* = 9) of the discovery cohort, and assessed analyte levels based on the median split of total TAM infiltration. Interestingly, in MGM tissues with high TAM infiltration, we discovered a significant increase of G-CSF, Eotaxin, IL-1β, IL-1ra, and IL-4 (Fig. 2a; *P* = 0.011 for G-CSF, *P* = 0.023 for Eotaxin,* P* = 0.010 for IL-1β, *P* = 0.008 for IL-ra, and *P* = 0.020 for IL-4), as well as a tendency toward increased levels of IL-6 (Fig. 2a; *P* = 0.064). Particularly, the cytokines IL-1β, IL-1ra, IL-4, and IL-6 are known to be TAM-associated, and both IL-4 and IL-6 are described in the literature as immunosuppressive cytokines that favor a tumor-supportive micromilieu [7]. When assessing cytokine levels based on WHO grading, we observed significantly increased protein levels in higher-grade tumors for a number of other factors, including the immunosuppressive cytokines IL-8 and IL-10, as well as the angiogenesis-promoting factor VEGF (Suppl. Fig. S4a) [2, 7, 13, 16]. Furthermore, in our PFS analysis of newly diagnosed patients, we found tendencies for inferior PFS in tumors with high levels of TAM-associated immunosuppressive cytokines IL-4 and IL-6 (Suppl. Fig. S4b-c).

To further elucidate the immune network in MGMs, we integrated the protein concentrations and TAM infiltration data from our present study with TIL infiltration data (relative infiltration of CD3^+^ TILs per TCC in MGM tissue in the same patient cohort) from our previous publication [49] into a correlation matrix for a sub-cohort of *n* = 46 cases (Fig. 2b, Suppl. Table S3-4). Calculating the gap statistic resulted in the identification of four distinct clusters (Suppl. Fig. S4d), characterized by varying compositions and sizes, and differing degrees of association with the other clusters. The largest cluster contained the pro-inflammatory cytokines IFN-γ and TNF-α as well as the factors Eotaxin, PDGF-BB, G-CSF, and the TAM-associated cytokines GM-CSF and IL-4. Interestingly, especially the pro-inflammatory cytokines IFN-γ and TNF-α strongly correlated with another smaller cluster of several pro-tumoral factors, which are well described in the literature to create an immunosuppressive niche including IL-6, IL-8, IL-10, and VEGF, and in addition IL12-p70, of which all five factors are also known to be secreted by TAMs in the local TME [2, 7, 13]. Furthermore, the last cluster was formed by recruiting chemokines including MCP-1 (CCL2), MIP-1α (CCL3), MIP-1β (CCL4), RANTES (CCL5) [43], and the cytokines IL-9, IL-1β, IL-1ra. IL-1β acts as a pleiotropic cytokine and has been shown to drive carcinogenesis and metastasis in the tumor context through various mechanisms [18, 21]. Importantly, the balance between IL-1β and its natural antagonist IL-1ra influences the tumor microenvironment's inflammatory status and impacts TAM polarization and activity [18, 21]. In our analysis, IL-1β and IL-1ra were also significantly correlated with the infiltration of TAMs and pro-tumoral M2-like TAMs within the first (cellular) cluster. In summary, quantification of 24 immune-related cytokines, chemokines, and growth factors revealed a complex, TAM-driven immunosuppressive microenvironment in MGMs, highlighting the role of TAMs in secreting immunosuppressive and tumor-supportive cytokines.

### High TAM infiltration is associated with poor progression-free survival outcome in meningioma patients and counteracts the beneficial effect of TILs

**Fig. 3:**
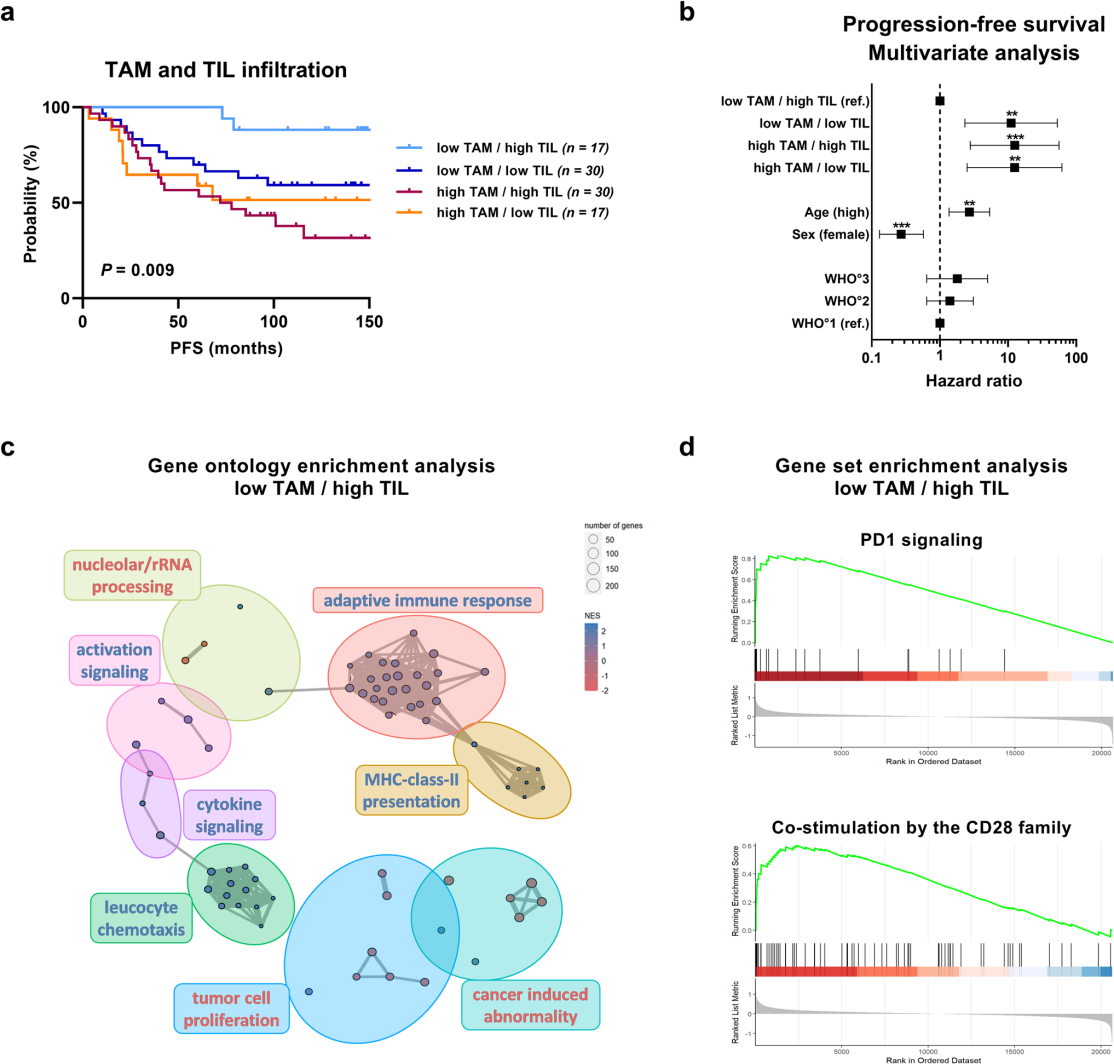
Impact of TAM and TIL infiltration on survival and transcriptional programs in meningiomas. **a** Kaplan–Meier plot showing PFS according to combined TAM (CD68^+^/TCC) and TIL (CD3^+^/TCC) infiltration in newly diagnosed MGMs of the discovery cohort. The patients were categorized into four groups according to their combined median TAM and TIL infiltration into (1) low TAM/high TIL (light blue), (2) low TAM/low TIL (dark blue), (3) high TAM/high TIL (dark red), and (4) high TAM/low TIL (orange) infiltration, respectively. **b** Multivariate analysis for PFS including TAM and TIL infiltration grouping, patient age, sex, and WHO grade. Statistical significance was calculated using log-rank test in (**a**) and Cox proportional hazard model in (**b**). **c–d** Gene expression analysis of microarray data [GSE74385 (*n* = 62 cases) [52] with additional *n* = 35 MGM cases] according to the combined median TAM and TIL infiltration in patients with newly diagnosed and recurrent MGMs. For the analysis, the low TAM/high TIL group was compared to the three remaining groups. **c** GO enrichment analysis in the low TAM/high TIL group. Down-regulated gene sets are depicted in red, while up-regulated gene sets are depicted in blue. **d** Reactome Pathway Database GSEA in the low TAM/high TIL group showing up-regulated PD1 signaling and up-regulated co-stimulation by the CD28 family. *GO* Gene ontology, *GSEA* Gene set enrichment analysis, *MGM* Meningioma, *NES* Normalized enrichment score, *PFS* Progression-free survival, *ref* Reference, *TAM* Tumor-associated macrophage, *TCC* Total cell count, *TIL* Tumor-infiltrating T lymphocyte. Statistical significance: *, *P* < 0.05; **, *P* < 0.01; ***, *P* < 0.001

To further elucidate the complex association between macrophages and T cells in MGMs and their distinct impact on PFS and transcriptional programs, we performed an integrative survival analysis of TAM infiltration and previously published TIL infiltration data in the same discovery cohort [49]. To this end, the above stated selection criteria (GTR, no prior treatment, follow-up > 5 years) were applied to the study sample to prevent any survival bias, resulting in a cohort of 94 patients with newly diagnosed tumors, which were then categorized into four groups according to their combined median TAM and TIL infiltration into (1) low TAM/high TIL, (2) low TAM/low TIL, (3) high TAM/high TIL, and (4) high TAM/low TIL infiltration, respectively (Fig. 3a, Suppl. Fig. S5a-c). Significant differences in PFS were observed among the four groups (Fig. 3a; *P* = 0.009). Patients with high TIL and low TAM infiltration exhibited superior outcomes, with a median PFS of 146.5 months (*n* = 17, light blue). In contrast, the group with low TAM and low TIL numbers (*n* = 30, dark blue) exhibited an intermediate survival rate, with a median PFS of 101.9 months. Notably, both groups with high TAM infiltration exhibited the most unfavorable outcomes. The group with high TAM and high TIL infiltration (*n* = 30, dark red) had a median PFS of 74.9 months and the group with high TAM and low TIL numbers (*n* = 17, orange) had an even worse outcome with a median PFS of 64.5 months. This is of particular interest as the group with high TAM and high TIL infiltration demonstrated comparable high TIL numbers to the group with the most optimal outcome, namely low TAM and high TIL infiltration (Suppl. Fig. S5b). These findings suggest that high TAM infiltration exerts the most dominant negative influence on patient outcome, counteracting the beneficial effect of TILs. Moreover, in a subsequent multivariate analysis, including clinically relevant covariates (age, sex, and WHO grade), we were able to show that high TAM and high TIL infiltration (Fig. 3b, Suppl. Table S5; HR = 12.48; *P* < 0.001) as well as high TAM and low TIL infiltration (Fig. 3b, Suppl. Table S5; HR = 12.42; *P* = 0.002) are independent prognostic factors for inferior PFS in patients with newly diagnosed MGMs. Additionally, we analyzed the TAM/TIL ratio as a Z-transformed continuous variable in a subsequent multivariate analysis, thereby confirming the combined TAM/TIL infiltration ratio as an independent prognostic factor (Suppl. Fig. S5d, HR = 1.35; *P* < 0.001).

Next, we aimed to explore the impact of the combined TAM and TIL infiltration on the transcriptional programs in MGM tissues by re-analyzing our microarray dataset (GSE74385 [52], *n* = 62 cases with additional *n* = 35 MGM cases). To increase statistical power, we used the complete dataset containing both newly diagnosed and recurrent tumors and performed gene expression analysis contrasting the low TAM and high TIL group with the three remaining groups. GO enrichment analysis to illustrate the superior biological process revealed several significantly shared GO terms in the low TAM/high TIL group, of which GO terms for tumor cell proliferation and cancer-induced abnormality were found to be down-regulated (in red; Fig. 3c, Suppl. Table S6), whereas GO terms for adaptive immune response, MHC class-II presentation, leukocyte chemotaxis, and cytokine signaling were found to be up-regulated (in blue; Fig. 3c; Suppl. Table S6), indicating an immunologically active microenvironment in tumors with low TAM/high TIL infiltration. In addition, GSEA using the Reactome Pathway Database revealed increased PD1 signaling (normalized enrichment score (NES) = 2.398, *P*_adj_ < 0.0001) and increased T-cell signaling represented through co-stimulation by the CD28 family pathway (NES = 2.173, *P*_adj_ < 0.0001) in MGMs with low TAM and high TIL infiltration (Fig. 3d), further supporting an immunostimulatory TME within these tumors.

Taken together, our gene expression analysis demonstrated TAM and TIL infiltration to be associated with significant changes in the transcriptional profiles of tumors. Further, high TAM infiltration, both in combination with low and high TIL numbers, was associated with inferior PFS in newly diagnosed MGMs and was confirmed as an independent prognostic factor in multivariate analysis. Thus, our data suggest that TAMs play a vital role in establishing and maintaining the immunosuppressive TME of MGMs suggesting a negative impact on patient outcome which counteracts the beneficial prognostic effects of TILs.

### Methylation-based deconvolution analysis confirms the dominant negative impact of TAM infiltration on patient survival in an independent validation cohort

**Fig. 4:**
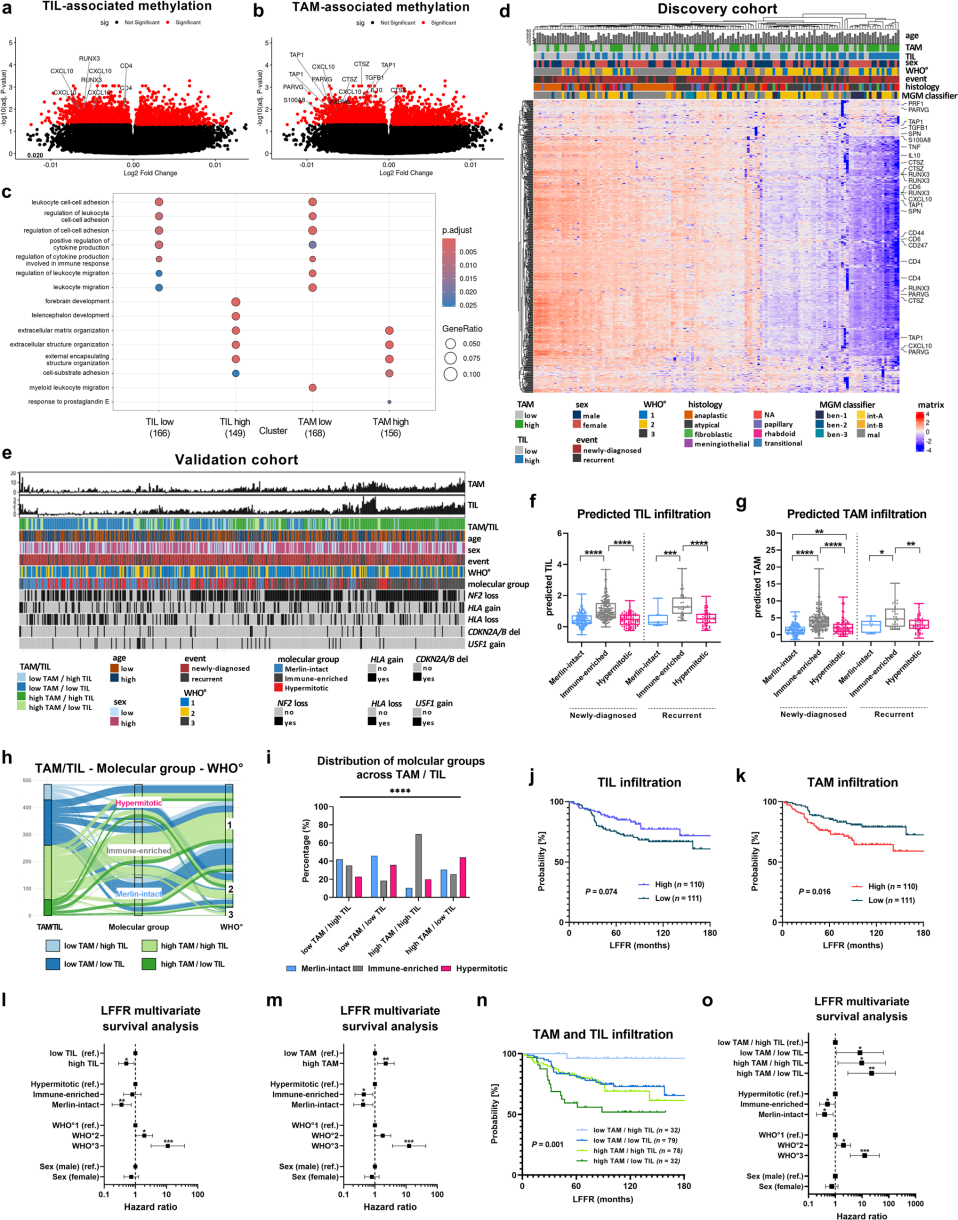
Methylation-based deconvolution predicts TAM and TIL infiltration and confirms their prognostic role in meningiomas. Volcano plot of **a** TIL- and **b** TAM-associated methylation highlighting significantly associated CpGs in red (*P*_*adj*_ > 0.05). **c** Dot plot showing GO terms associated with differentially methylated CpGs for TIL and TAM. **d** Heatmap of discovery cohort (Heidelberg, *n* = 144) showing differentially methylated CpGs (*n* = 320 with lower methylation) with clinical and experimental parameters. **e** Oncoprint of validation cohort (UCSF/HKU, *n* = 485) showing predicted TAM and TIL infiltration as well as clinical experimental parameters. **f-g** Predicted infiltration according to molecular groups for newly diagnosed and recurrent MGMs of (**f**) TILs and (**g**) TAMs. **H** Sankey diagram showing associations of predicted TAM/TIL groups, molecular groups, and WHO grading. **i** Bar plot showing distribution of molecular groups across predicted TAM/TIL groups. **j** Kaplan–Meier plot for LFFR based on high (purple curve) and low (turquoise curve) predicted TIL infiltration in newly diagnosed MGMs (*n* = 221). **k** Kaplan–Meier plot for LFFR based on high (red curve) and low (turquoise curve) predicted TAM infiltration in newly diagnosed MGMs. **l-m** Multivariate survival analysis for LFFR including prognostic confounders (sex, WHO grade, molecular group) with (**l**) predicted TIL infiltration and (**m**) predicted TAM infiltration. **n** Kaplan–Meier plot showing LFFR according to predicted and combined TAM and TIL infiltration in newly diagnosed MGMs. Patients were categorized into four groups according to their combined median TAM and TIL infiltration into (1) low TAM/high TIL (light blue), (2) low TAM/low TIL (blue), (3) high TAM/high TIL (light green), and (4) high TAM/low TIL (green) infiltration, respectively. **o** Multivariate analysis for LFFR including predicted TAM and TIL infiltration grouping, patient sex, molecular group, and WHO grade. *ben* Benign, *int* Intermediate, *GO* Gene ontology, *HKU* University of Hong Kong, *LFFR* Local freedom from recurrence, *mal* Malignant, *MGM* Meningioma, *NA* Not available, *R* Recurrent, *ref* Reference, *TAM* Tumor-associated macrophage, *TIL* Tumor-infiltrating T Lymphocyte, *UCSF* University of California San Francisco. Statistical significance: *, *P* < 0.05; **, *P* < 0.01; ***, *P* < 0.001; ****, *P* < 0.0001

To validate our findings on the prognostic role of TAMs and TILs in MGMs from the discovery cohort, we analyzed an independent MGM validation cohort provided by the University of California San Francisco (UCSF) and the University of Hong Kong (HKU), originally published by Choudhury et al. [12], including *n* = 565 meningioma specimens spanning all WHO grades (*n* = 388 WHO grade 1, *n* = 142 WHO grade 2, *n* = 35 WHO grade 3, Suppl. Table S7; GSE183647). To this end, we developed a DNA methylation-based machine learning approach to predict infiltration rates of TILs and TAMs. Initially, we leveraged our directly assessed TAM and TIL infiltration data to identify differentially methylated CpG sites associated with these immune cells in the corresponding methylation data while controlling for WHO grade effects. This analysis revealed a total of 639 differentially methylated CpGs (*P*_*adj*_ < 0.05), with 315 sites linked to TILs and 324 to TAMs (Fig. 4a-b). Annotation of these CpG sites to their corresponding genes highlighted several immune-related markers, such as CD4 (TILs) and CXCL10 (TAMs and TILs), which exhibited lower methylation levels (Fig. 4a-b). Subsequent GSEA demonstrated that CpG sites with reduced methylation were enriched in immune-related pathways (e.g., leukocyte cell–cell adhesion/migration), whereas those with increased methylation were associated with tumor-related pathways (e.g., Figure 4c). Therefore, only CpG sites with lower methylation (*n* = 320) were entered in the machine learning pipeline. Using elastic-net regression on the Heidelberg discovery cohort (*n* = 144 cases; heatmap shown in Fig. 4d with *n* = 320 CpG sites with lower methylation), we minimized collinearity and selected the most informative features, ultimately identifying 63 CpGs for TILs and 43 for TAMs (Suppl. Fig. S6a-b, Suppl. Table S8) as DNA methylation-based signatures for predicting immune cell infiltration. Validation in the Heidelberg cohort confirmed strong correlations between observed and predicted immune cell infiltration (r = 0.8390, *P* < 0.0001 for TILs; r = 0.7935, *P* < 0.0001 for TAMs; Suppl. Fig. S6c-d), supporting the robustness of our model. The pretrained regression-based deconvolution model was then applied to the larger external UCSF/HKU methylation cohort, enabling the independent prediction of TIL and TAM infiltration in a subset of 533 samples (Suppl. Fig. S6e). Next, we examined the association between the predicted immune cell infiltration and comprehensive molecular as well as clinical data, including molecular groups (Merlin-intact, Immune-enriched, Hypermitotic), genetic alterations (*NF2* loss, *HLA* gain/loss, *CDKN2A/B* deletion, *USF1* gain on chr1q), patient age, sex, tumor status (newly diagnosed vs. recurrent), and WHO grade in *n* = 485 cases (Fig. 4e) [12]. This analysis revealed that both TAM and TIL infiltration were highest in the Immune-enriched molecular group across newly diagnosed and recurrent MGMs (*, *P* < 0.05; **, *P* < 0.01; ***, *P* < 0.001; ****, *P* < 0.0001; Fig. 4f-g). In contrast, predicted TAM infiltration was lowest in the Merlin-intact group and significantly elevated in the Hypermitotic group, a pattern not seen for TILs. These observations not only validate our DNA methylation–based deconvolution approach but also highlight an enrichment for TAMs in groups associated with poorer outcomes compared to the Merlin-intact group (Suppl. Fig. S6f). Further analysis of the associations among predicted TAM/TIL groups, molecular subtypes, and WHO grades showed a broad distribution across categories (Fig. 4h). Notably, when evaluating molecular group distributions, we found significant differences between TAM/TIL categories (****, *P* < 0.0001; Fig. 4i): the high TAM/low TIL group contained the highest proportion of clinically aggressive Hypermitotic MGMs, whereas groups with high TIL infiltration showed the lowest proportions of this adverse molecular subtype. Further, groups with low TAM infiltration demonstrated the highest proportions of the clinically more favorable Merlin-intact MGMs.

For subsequent survival analyses, we focused on a rigorously selected sub-cohort of 221 newly diagnosed MGM patients, who had undergone GTR and had a minimum follow-up of 36 months (Suppl. Table S9). Patients were stratified by median infiltration values into high- and low-infiltration groups for both TILs (median predicted infiltration of 0.64%/TCC) and TAMs (median predicted infiltration of 2.58%/TCC). We observed a trend toward improved local freedom from recurrence (LFFR) for patients with high TIL infiltration (*P* = 0.074; Fig. 4j), while high TAM infiltration was significantly associated with inferior recurrence-free survival (LFFR; *P* = 0.016; Fig. 4k). Multivariate analyses, accounting for sex, WHO grade, and molecular group, further identified high TIL infiltration as an independent predictor of improved LFFR and high TAM infiltration as an independent predictor of worse LFFR in newly diagnosed MGMs (*, *P* < 0.05; **, *P* < 0.01; Fig. 4l-m, Suppl. Table S10–11). These results are consistent with our initial findings based on tissue cytometry analyses in the discovery cohort (Fig. 1h-i) [49].

Finally, our analysis of combined TAM and TIL infiltration in the validation cohort revealed significant LFFR differences among the four defined groups (*P* = 0.001; Fig. 4n). Patients in the low TAM/high TIL group exhibited superior outcomes with a median LFFR of 86.5 months (*n* = 32, light blue), whereas those with high TAM and low TIL infiltration had the poorest outcomes (median LFFR of 65.4 months, *n* = 32, green). Intermediate PFS rates were observed in the low TAM/low TIL (median LFFR 99.4 months, *n* = 79, blue) and high TAM/high TIL (median LFFR 77.2 months, *n* = 78, light green) groups. Multivariate analysis incorporating key clinical covariates (sex, WHO grade, and molecular group; Fig. 4o; Suppl. Table S12) confirmed that the low TAM/low TIL and high TAM/high TIL profiles were independent predictors of inferior LFFR (low TAM/low TIL: HR = 8.38, *P* < 0.05; high TAM/high TIL: HR = 9.69, *P* < 0.05) as well as the high TAM/low TIL group showing the highest HR of 22.80 (*P* < 0.01; Fig. 4o; Suppl. Table S12). These findings emphasize the beneficial prognostic impact of high TIL levels and the deleterious effect of high TAM infiltration in MGMs, particularly in the absence of T cells.

In summary, the application of our refined DNA methylation–based deconvolution approach to a second, independent, and large MGM study cohort [12] robustly validates our earlier observations on the opposing prognostic roles of TILs and TAMs in MGMs. These results underscore the importance of understanding the tumor immunobiology to inform future immunotherapeutic development and clinical decision-making.

## Discussion

TAMs represent the main immune cell population in several solid cancer entities, where they are strongly implicated in tumor development and progression [9, 13], which has also been extensively studied in brain malignancies [42]. Due to the comparably lower frequency of clinically aggressive MGMs and due to the lack of long-term survival data from patients in most studies, there is still insufficient knowledge about the prognostic role of TAMs in this tumor entity. To address this, we first investigated TAM and pro-tumoral M2-like TAM infiltration in a discovery cohort of 195 clinically well-annotated cases to evaluate their prognostic value on long-term patient survival (PFS). We discovered a highly heterogeneous but substantial total TAM infiltration (CD68^+^ cells/TCC), which was four times higher than for TILs (CD3^+^ cells/TCC) in the same large patient cohort of newly diagnosed MGMs [49]. We further found overall higher numbers of TAMs and immunosuppressive M2-like TAMs in clinically aggressive MGMs as well as in tumors of male and older patients. Overall, high TAM infiltration turned out as an independent prognostic factor for poor PFS outcome in MGM patients dominating over the opposing beneficial prognostic effect of TIL infiltration. Furthermore, we were able to validate these findings in an independent study cohort compromising *n* = 485 MGM specimens by predicting macrophage and T-cell infiltrates following a DNA methylation-based deconvolution approach, which may be of potential use to determine TAM/TIL ratios in future clinical trials as MGM-specific immune-related biomarkers. Thus, our data provide strong evidence for the pro-tumoral state of TAMs in MGMs and their dominant impact on disease progression and patient outcome.

Noteworthy, a major strength of this work is our prognosis-focused integrative multiomics approach utilizing two large independent study cohorts with a total number of *n* = 680 MGM specimens, covering *n* = 510 newly diagnosed and *n* = 170 recurrent tumors, as well as combining wet-lab and computational methodologies to explore TAM and TIL frequencies. In addition, our tissue cytometry workflow for assessing TAM numbers and their polarization state in whole-tissue sections (median area of ~ 10.9 mm2) rather than the use of small tissue microarrays (TMAs; core size of 0.36–4.00 mm2) allows for a highly reliable quantitative analysis, and reduces overall bias from small regional variability. Even though we observed small differences in numbers of TAM infiltration between subsampled areas of large MGM specimens, notably, the overall categorization of MGMs into “TAM high” or “TAM low” tumors remained unchanged, which is linked to patient prognosis. Moreover, we addressed the issue of regional variability in our additional transcriptome and methylome analyses using bulk tumor tissue. Importantly, these analyses corroborated the results on immune cell infiltration from our tissue cytometry analyses for TAMs as well as for TILs [49].

A number of other studies has investigated the myeloid cell compartment and reported the presence of TAMs in MGMs before [1, 14, 24, 27, 44, 46, 61–64]. However, most of these studies had significant limitations in their study design, e.g., they investigated relatively small and unbalanced study cohorts with low numbers of clinically aggressive tumors and/or without clinical follow-up and subsequent PFS analysis [1, 14, 46, 61–63]. In 2021, Yeung and colleagues examined the infiltration of TAMs among other immune cell subsets in a cohort of 73 MGM specimens (*n* = 56 WHO grade 1, *n* = 13 WHO grade 2, and *n* = 4 WHO grade 3) using multicolor immunofluorescence stainings on TMA-based small biopsies [61]. Similar to our findings, their analysis revealed a heterogeneous TAM infiltration in MGMs with the majority of TAMs displaying a pro-tumoral M2-like phenotype (CD68^+^ CD163^+^ cells). However, in contrast to our data, the authors reported no significant differences in TAM and pro-tumoral TAM infiltration rates across WHO grades, which could be due to differences in study design (staining of biopsy-based TMAs vs. here whole-tissue sections) and overall limited number of higher-grade tumors (*n* = 13 WHO grade 2/3).

In a recent study, Zhang and colleagues also performed multicolor immunofluorescence stainings and reported TAM (CD68^+^ cells) and diverse TAM subpopulation infiltration rates according to the methylation classes (MCs) from the Heidelberg classifier [51] in a rather small sub-cohort of *n* = 31 MGMs (*n* = 17 MC benign-1/-2/-3; *n* = 6 MC intermediate-A/B; *n* = 8 MC malignant) with limited higher-grade samples in the total cohort (*n* = 35 WHO grade 1; *n* = 10 WHO grade 2; *n* = 4 WHO grade 3). Of note, while we stained more broadly for CD68 + cells, which is primarily expressed by macrophages, monocytes, and microglia, Zhang et al. also differentiated between macrophages (CD68 + TMEM119- P2RY12-) and microglia cells (CD68 + TMEM119 + P2RY12 +) as TAM subpopulations (all CD68 + cells). In their analysis, proportions of pro-tumoral M2-like TAMs (% within total TAMs) composed the majority of total TAMs (> 75%) across WHO grade and all presented MCs [64], which is in line with our results showing that MGM-TAMs display mainly a pro-tumoral phenotype. On a functional level, Zhang and colleagues were also able to demonstrate the pro-tumoral activity of M2-polarized TAMs on MGM. They developed a patient-derived 3D in vitro co-culture model and observed significantly increased MGM tumor cell proliferation over time in the presence of M2-polarized macrophages when compared to untreated tumor spheroids. In addition, analysis of macrophage-related cytokine expression revealed an association between high *IL6* expression and tumor recurrence in patients of all WHO grades [64]. Notably, these findings corroborate our cytokine analysis, demonstrating that higher IL-6 levels in MGM tissue are associated with high TAM infiltration and inferior outcome of patients in newly diagnosed tumors.

As a major strength, in contrast to these previous studies, we present long-term PFS data with a minimum follow-up of 5 years, enabling us to investigate longitudinal changes of TAM composition in MGM as well as their impact on patient outcome in our discovery cohort. Thus, to the best of our knowledge, this is the first comprehensive report demonstrating not only TAM frequencies and their polarization state but also their survival-associated changes in a large study cohort composed of newly diagnosed as well as recurrent MGMs containing also high frequencies of higher-grade tumors. In our analysis, we identified high TAM infiltration to be associated with inferior PFS in newly diagnosed MGM patients, while in recurrent MGMs, we observed no further deterioration of survival in association with TAM infiltration. In addition, we discovered high M2-like TAM infiltration as a prognostic factor for poor patient outcome independent of other prognostic confounders, such as age, sex, and WHO grade. Moreover, by integrating our previously published data on TIL infiltration [49] in MGM in the same patient cohort, we analyzed the combined TAM and TIL infiltration and identified high TAM infiltration as the dominant negative prognostic factor on patient outcome in MGM tissue. Noteworthy, the two study cohorts we have analyzed differed regarding distribution of WHO grades, tumor event, age, and sex. While the discovery cohort had a higher proportion of clinically aggressive tumors, the validation cohort represented the clinical prevalence with more benign tumors, which was also displayed with respect to age and sex, e.g., a higher female-to-male ratio in the validation cohort than in the discovery cohort (2.1 vs. 1.6 for newly diagnosed tumors). However, despite these differences, we were able to demonstrate the prognostic role of TAMs, TILs, and their combined infiltration in both study samples in our analyses, comprising altogether *n* = 680 MGM specimens.

Moreover, the predictions of TAM and TIL numbers in the validation cohort using a well-refined DNA methylation-based deconvolution analysis enabled us to first report on associations between WHO classification, molecular groups, and TAM/TIL categories for this tumor type. Interestingly, we found the highest proportion of the Hypermitotic MGMs, presenting the clinically most aggressive molecular group, in the high TAM/low TIL group, whereas the proportion of Hypermitotic MGMs was similarly small in both the high TAM/high TIL and low TAM/high TIL categories. As expected, the highest proportion of Immune-enriched MGMs was found in the high TAM/high TIL group, but was also distributed across the remaining TAM/TIL categories and second largest in the low TAM/high TIL group. These results underline the complex role and interplay of different immune infiltrates in MGMs as well as their influence on tumor behavior and disease progression, and further emphasize the need to differentiate between diverse immune cell subsets to distinguish between favorable and unfavorable Immune-enriched MGM subtypes.

Altogether, the deconvolution analysis represents a remarkable pillar of our study, validating our initial findings from the discovery cohort as well as highlighting the important role of the immune microenvironment in MGM subtypes across molecular groups. Furthermore, with our computational approach, we provide a pipeline to deconvolve TAM and TIL infiltration numbers from MGM-derived DNA methylation data by the help of our specific CpG signatures (see Supplementary Table S7), which can be applied to existing and newly generated methylation data. Importantly, MGMs are highly eligible to DNA methylation profiling (DNAMP), which provides a valuable diagnostic tool for superior tumor typing in comparison to standard histopathological analyses and thus, if applied, can improve the management of clinically more aggressive tumor subtypes [50]. DNAMP is a well-established and well-recognized analysis for MGM risk profiling and multiple MGM classifiers have been developed [3, 12, 37, 40, 51], and were further evolved with mutational profiles, gene expression data, as well as histological features into multi-layered integrative classification systems for clinical applications [11, 12, 30, 36, 58]. Despite their differences, DNA methylation data are available from all these classifiers and are ready-to-use for deconvolving immune cell fractions in sizable MGM cohorts. Therefore, we emphasize the use of our MGM-specific deconvolution analysis as well as the need to integrate the tumor’s immunobiology much more closely into therapy development and clinical decision-making to ultimately achieve a holistic research and treatment approach.

Based on our findings, we also promote TAMs and M2-like TAMs as potential therapeutic targets for immunotherapeutic approaches in MGM patients. In contrast, clinical trials have so far focused primarily on T-cell-based immune checkpoint inhibition, despite evidence that MGMs often lack key prerequisites for ICB success, which are high tumor mutational burden, high T-cell infiltration, and ICB target expression [56, 59]. Accordingly, it is no surprise that results from clinical trials targeting the PD1/PD-L1 axis or CTLA4, either as mono- or combination therapy, have been fairly disappointing in MGM patients [4, 6, 19, 28, 55, 59]. Fortunately, novel immunotherapeutic strategies have entered pre-clinical and clinical testing in recent years and have focused among others on targeting immunosuppressive macrophages at the tumor site [9, 10, 45]. TAMs as treatment targets have attracted great interest, especially in brain malignancies, such as gliomas, which are also characterized by a low tumor mutational burden, lower infiltration of T cells but higher numbers of TAMs [20, 31, 42]. In 2021, Yeung and colleagues were the first to investigate a macrophage-targeting approach in an immune-competent syngeneic mouse model of MGM and reported anti-CSF1/CSF1R immunotherapy to be efficacious at inhibiting tumor growth [62], which has previously been reported to elicit anti-tumoral responses in other brain tumor models as well [15, 47, 60]. As a consequence, we further encourage to rethink immunotherapeutic approaches for MGM patients [55]. Moreover, we strongly suggest the use of our TAM- and TIL-associated CpG signatures to deconvolve immune cell infiltrates from bulk tumor DNA methylation data and their use as novel biomarkers in future ICB-based or macrophage-targeting clinical trials to enable the stratification of patients into treatment groups according to the tumor’s specific immune microenvironment. Finally, with regard to our matched pair and survival analysis in recurrent MGMs, where the presence of TAMs had no further impact on PFS, we additionally propose to consider the timing of immunotherapeutic approaches in MGM patients. In the past, clinical trials have primarily enrolled patients in an advanced disease stage, with recurrent tumors that have been heavily pre-treated and therefore likely have a highly immunosuppressive microenvironment, in which immunotherapeutic interventions, regardless of the target, may ultimately fail to induce clinically meaningful responses. Therefore, future clinical trials of immunotherapy, targeting macrophages in MGM, should be favorably given in the primary setting rather than at recurrence and could be eventually combined with radiotherapy or other T-cell-based immunotherapies to improve overall patient outcome.

In summary, we conducted a comprehensive prognostic role-oriented analysis of TAM and TIL infiltration, immune-related cytokines, and gene expression in a large, clinically well-annotated discovery cohort (*n* = 195) of both newly diagnosed and recurrent MGMs, including a substantial number of higher-grade tumors. Thereby, we found high M2-like TAM frequencies to be associated with inferior PFS in MGM patients, and importantly, identified high TAM infiltration as an independent prognostic factor for poor patient outcome, dominating the opposing beneficial prognostic effect of TILs by creating an immunosuppressive niche. Moreover, by following a sophisticated DNA methylation-based deconvolution approach to predict infiltration rates of TAMs as well as TILs in a second independent validation cohort (*n* = 485), we were able to confirm our findings on the prognostic roles of T cells and macrophages for MGM behavior and disease progression. Altogether, our data highlight an important role of immunosuppressive TAMs on tumor aggressiveness, and thus, we strongly suggest TAMs as attractive treatment targets for MGM immunotherapy. Furthermore, we strongly encourage the use of our methylation-based deconvolution approach to predict TAM and TIL infiltration rates as novel MGM-specific biomarkers for the next generation of immunotherapeutic clinical trials for MGM patients, hopefully leading to novel therapeutic breakthroughs in the near future.

## Supplementary Information

Below is the link to the electronic supplementary material.

Supplementary file1 (DOCX 4080 KB)

Supplementary file2 (XLSX 74642 KB)

## Data Availability

All data are being securely held within the Division of Experimental Neurosurgery, Department of Neurosurgery at the University Hospital of Heidelberg, Germany. All data have been systematically cataloged and are available from the corresponding author (CHM) upon reasonable request. The methylation dataset of the discovery cohort (Heidelberg) is available upon reasonable request from the Department of Neuropathology (FS) at the University Hospital of Heidelberg, Germany. Values for all data points in graphs are reported in the Supporting Data Values file. The microarray dataset is available in the GEO repository, GSE74385, [https://www.ncbi.nlm.nih.gov/geo/query/acc.cgi?acc = GSE74385] (https:/www.ncbi.nlm.nih.gov/geo/query/acc.cgi?acc = GSE74385). The methylation dataset of the validation cohort (UCSF/HKU) is available in the GEO repository, GSE183647 [https://www.ncbi.nlm.nih.gov/geo/query/acc.cgi?acc = GSE183647] (https:/www.ncbi.nlm.nih.gov/geo/query/acc.cgi?acc = GSE183647).
