# Supplementary material for: Tumor-associated macrophages in meningiomas: a novel biomarker for poor survival outperforming the benefits of T cells
Source: Acta Neuropathol. 2025 Oct 9;150(1):41. doi: 10.1007/s00401-025-02948-6 (PMC12511222; doi:10.1007/s00401-025-02948-6)
Supplement: Supplementary file 1 — Supplementary file1 (DOCX 4080 KB) [file 401_2025_2948_MOESM1_ESM.docx]

**SUPPLEMENTAL MATERIAL**

**SUPPLEMENTARY FIGURES**


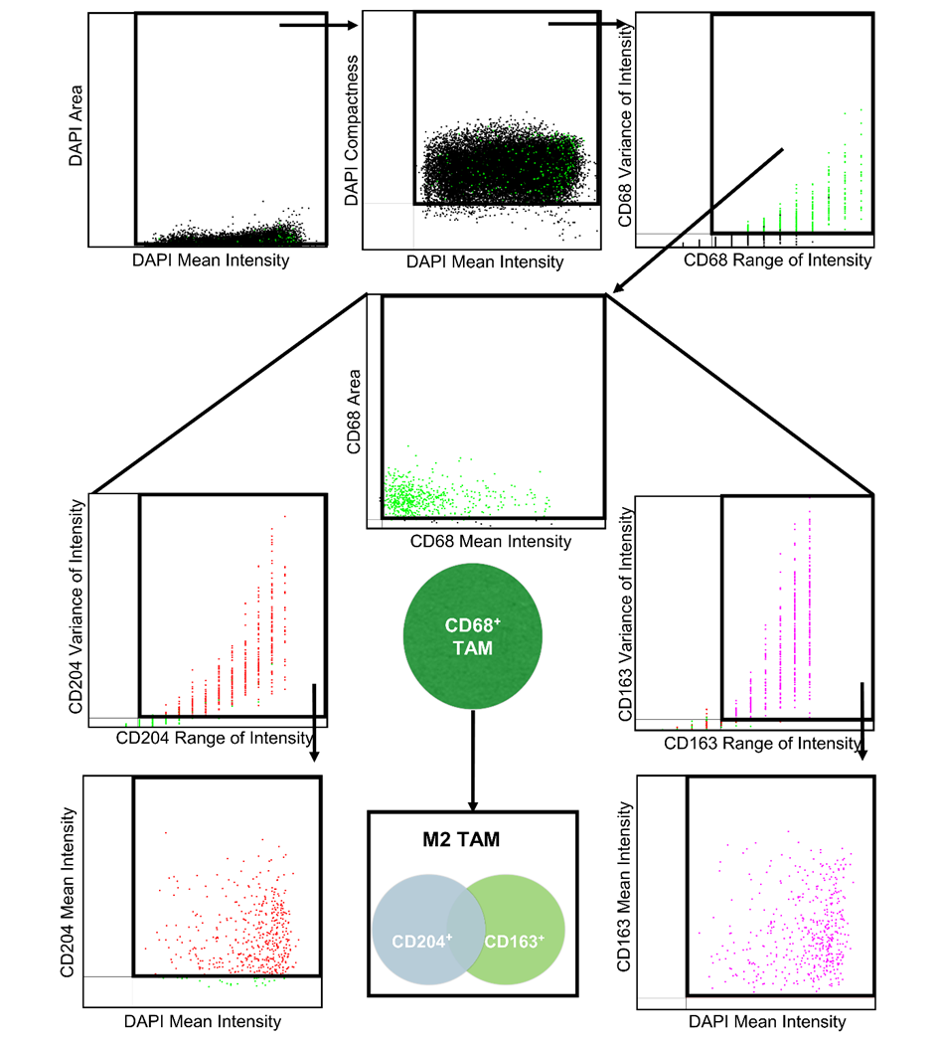


### Supplementary Figure S1: Workflow for tissue cytometry-based analysis.

Automated cell detection was based on DAPI. Irregular and extreme small and large DAPI were excluded at first. Subsequent detection of marker CD68 was conducted within the gate of the selected DAPI. Before the assessment to the three macrophage markers, unspecific homogenous background and dotted artifacts were filtered by the setting of area as well as variance of strength of fluorescence, respectively. CD68 positive cells were recognized as general TAM. Analysis of CD204 and CD163 were performed within the gate of CD68 positive cells. CD68^+^ cells with staining for CD204^+^ and/or CD163^+^ were recognized as pro-tumoral M2-like TAMs. Abbreviations: TAM, tumor-associated macrophage.


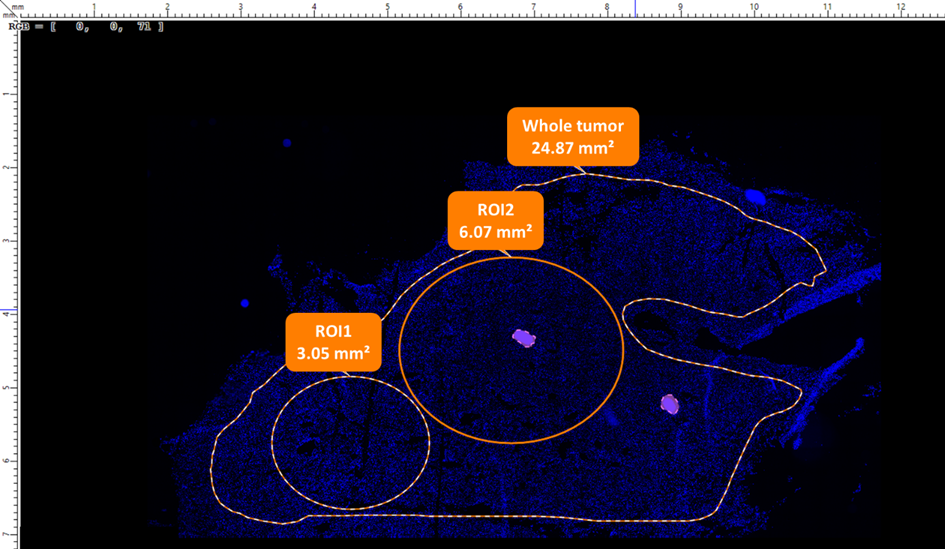


### Supplementary Figure S2: Meningioma tissue specimen with different sized areas (DAPI staining).

Exemplary case showing different sized areas / regions of interest (ROIs): ~3mm² (ROI1), ~6mm² (ROI2), and the whole tumor section (24.87mm²), which were analyzed analogously in tissue cytometry-based analysis to determine intra-tumoral regional variability.


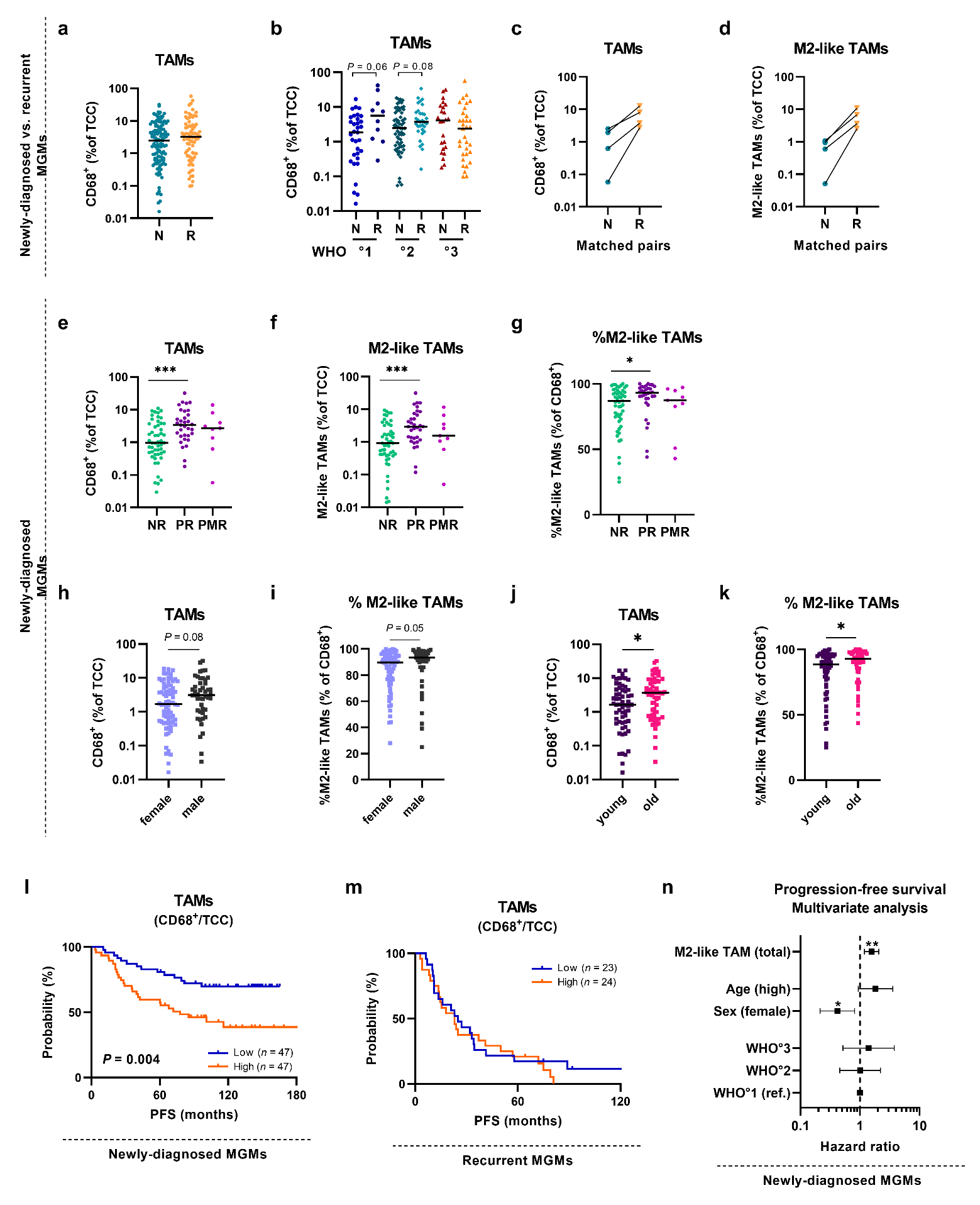


### Supplementary Figure S3: TAM infiltration and impact on survival in newly-diagnosed and recurrent meningiomas of the discovery cohort.

**a** TAM infiltration (CD68^+^/TCC) in newly-diagnosed (N) and recurrent (R) MGMs. **b** TAM infiltration across WHO grades in newly-diagnosed (N) and recurrent (R) MGMs. **c-d** Infiltration in newly-diagnosed (N) and recurrent (R) MGMs of matched pairs of (**c**) TAMs and (**d**) pro-tumoral M2-like TAMs (M2-like TAMs/TCC). **e-g** Infiltration in newly-diagnosed MGMs including non-recurring (NR), prospectively recurring (PR) and prospectively malignant-recurring (PMR) tumors of (**e**) TAMs, (**f**) M2-like TAMs and (**g**) proportions of M2-like TAMs (%M2-like TAMs/CD68^+^).  **h** TAM infiltration in MGMs of female and male patients in newly-diagnosed MGMs. **i** Proportions of M2-like TAM infiltration in MGMs of female and male patients in newly-diagnosed MGMs. **j-k** Infiltration in newly-diagnosed MGMs of young and old patients (median age of 60.8y) of (**j**) TAMs and (**k**) proportions of M2-like TAMs. **l-m** Kaplan-Meier plot for PFS based on high (orange curve) and low (blue curve) TAM infiltration in (**l**) newly-diagnosed and (**m**) recurrent MGMs. **n** Multivariate survival analysis for PFS including prognostic confounders (age, sex, WHO grade) and M2-like TAM infiltration (as a Z-transformed continuous variable). Statistical significance was calculated using Mann-Whitney-U test in (a-b, e-k), Wilcoxon matched-pairs signed rank test in (c-d), log-rank test in (l-m), and Cox proportional hazard model in (n). Abbreviations: MGM, meningioma; N, newly-diagnosed; NR, non-recurring; PMR, prospectively malignant-recurring; PR, prospectively recurring; PFS, progression-free survival; R, recurrent; ref, reference; TAM, tumor-associated macrophage; TCC, total cell count; y, years. Statistical significance: *, *P*<0.05; **, *P*<0.01; ***, *P*<0.001.


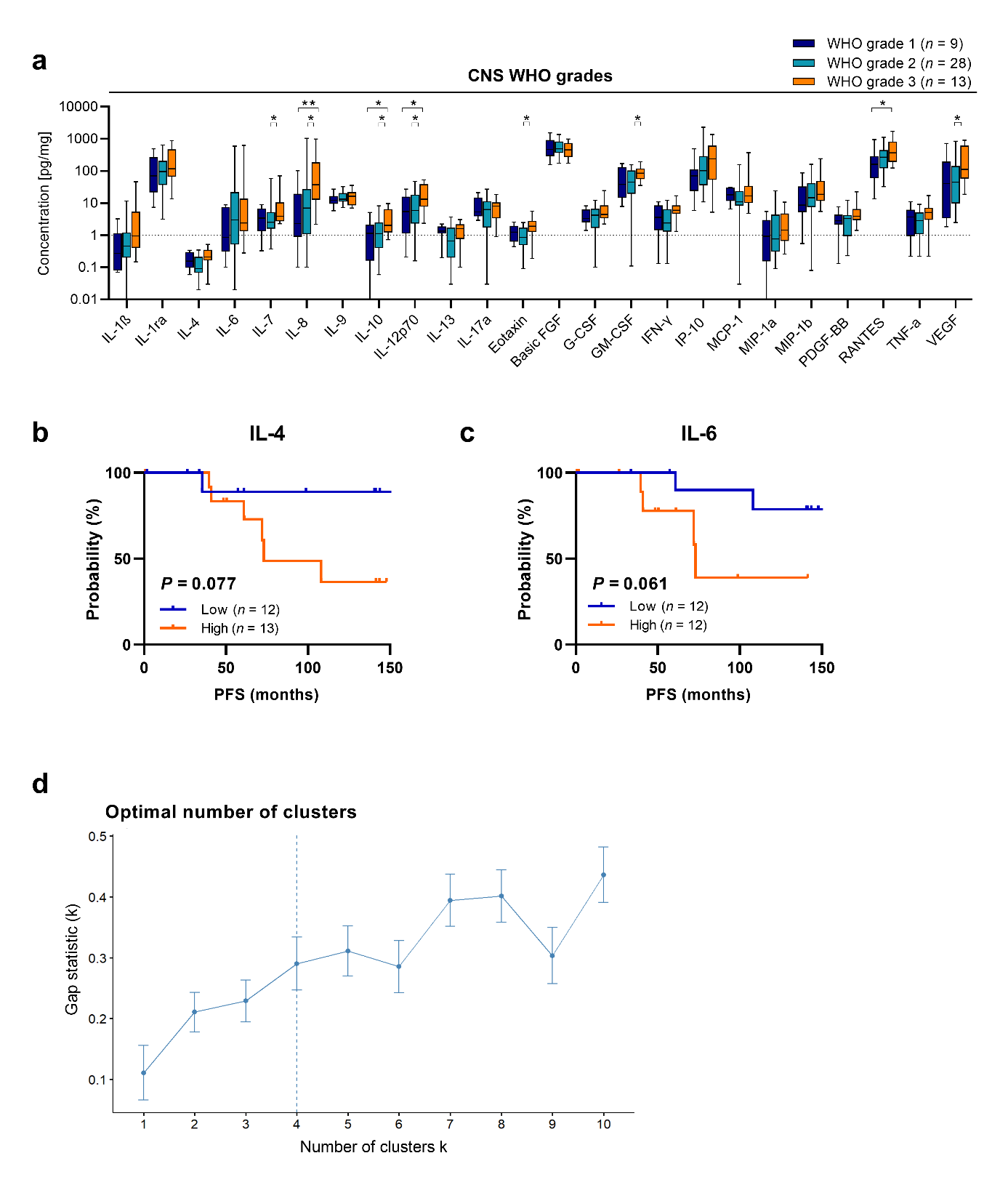


### Supplementary Figure S4: Cyto- and chemokine analysis of meningiomas.

**a** Concentrations of 24 cytokines and chemokines in MGM tissues (*n*=46, subset of discovery cohort) assessed by Luminex analysis comparing WHO grades. **b-c** Kaplan-Meier plot for PFS based on high (orange curve) and low (blue curve) cytokine levels in newly-diagnosed MGMs for (**b**) IL-4 and (**c**) IL-6. **d** Gap statistic to determine the optimal number of clusters in the correlation matrix. Statistical significance was calculated using Mann-Whitney-U test in (a) and log-rank test in (b-c). Abbreviations: MGM, meningioma; PFS, progression-free survival. Statistical significance: *, *P*<0.05; **, *P*<0.01.


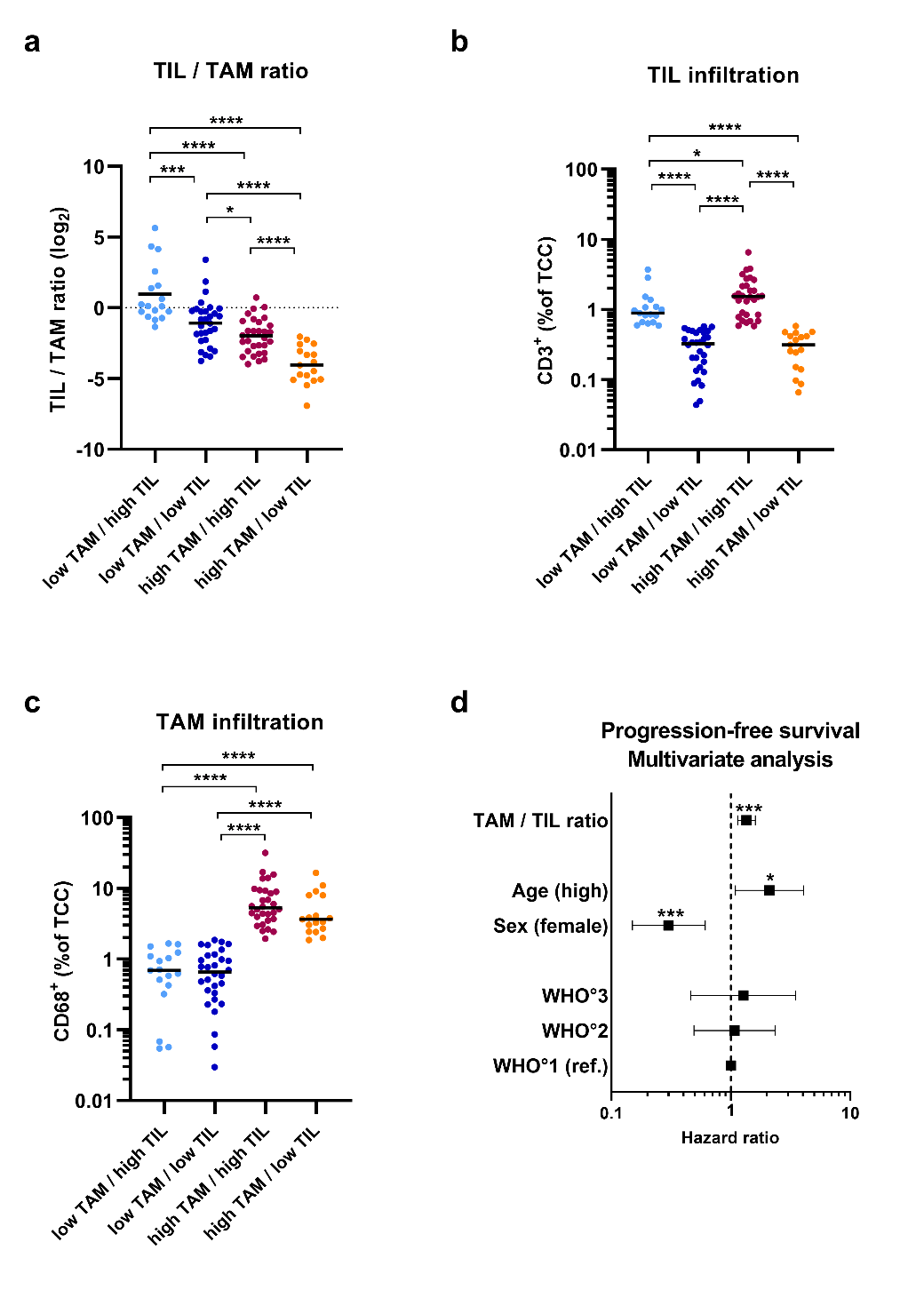


### Supplementary Figure S5: TAM and TIL infiltration in newly-diagnosed meningiomas of the discovery cohort.

**a-c** TAM and TIL infiltration in newly-diagnosed MGMs showing (**a**) TIL/TAM ratio, (**b**) TIL infiltration (CD3^+^/TCC) and (**c**) TAM infiltration (CD68^+^/TCC) across the four specified groups of patients according to their median TAM and TIL infiltration: (1) low TAM/high TIL (light blue), (2) low TAM/low TIL (dark blue), (3) high TAM/high TIL (dark red), (4) high TAM/low TIL (orange) infiltration, respectively. Lines show the mean in (a) and the median in (b-c). **d** Multivariate survival analysis for PFS including prognostic confounders (age, sex, WHO grade) and TAM/TIL ratio (as a Z-transformed continuous variable). Statistical significance was calculated using Student’s unpaired t test in (a), Mann-Whitney-U test in (b-c), and Cox proportional hazard model in (d). Abbreviations: ref, reference; TAM, tumor-associated macrophage; TCC, total cell count; TIL, tumor-infiltrating T-lymphocyte. Statistical significance: *, *P*<0.05; **, *P*<0.01; ***, *P*<0.001; ****, *P*<0.0001.


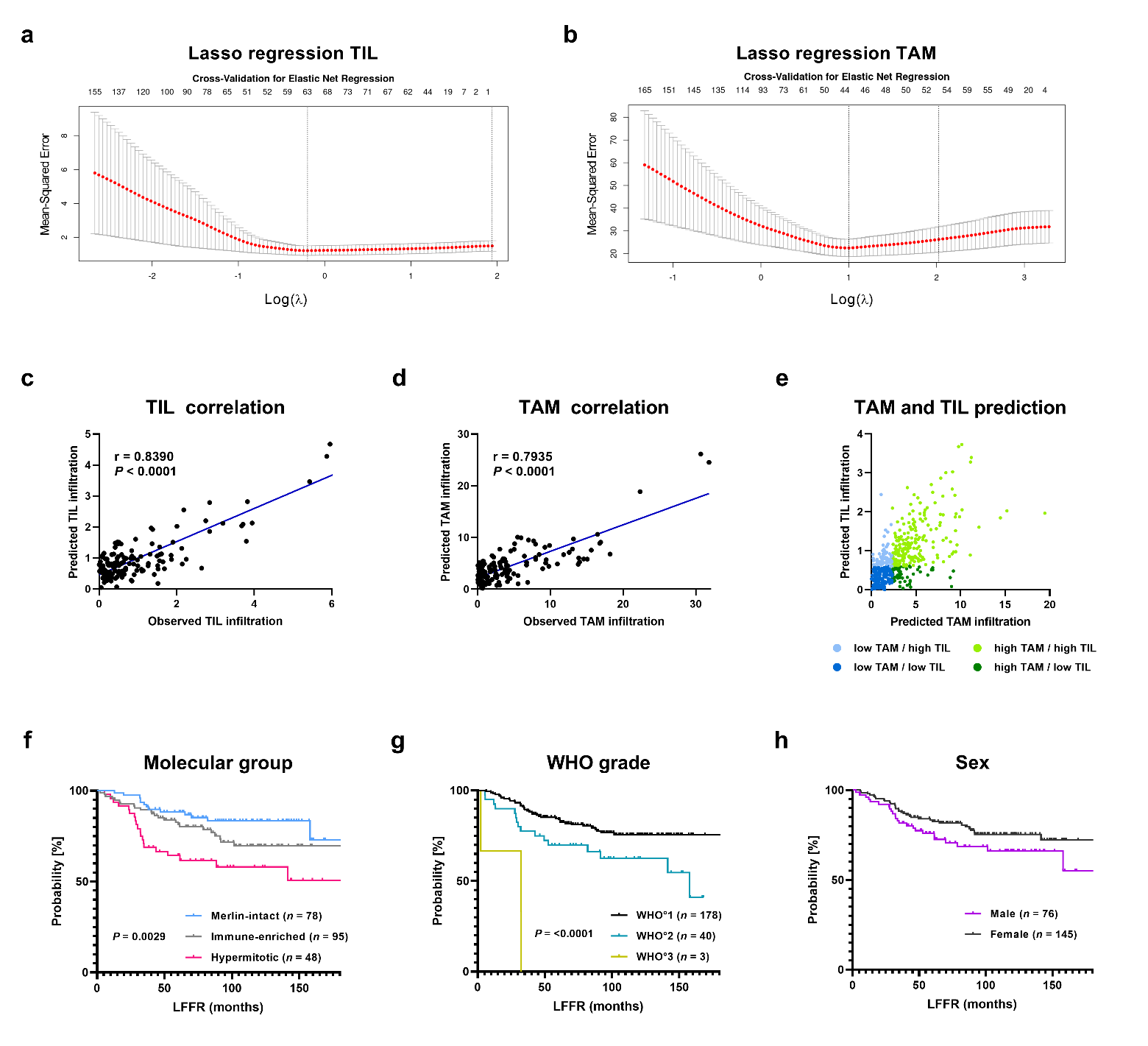


### Supplementary Figure S6: TAM and TIL prediction in meningioma discovery and validation cohort.

**a-b** Lasso regression model for (**a**) TIL and (**b**) TAM prediction in discovery cohort (Heidelberg, *n*=144). **c-d** Pearson correlation analysis showing association between observed and predicted (**c**) TIL and (**d**) TAM infiltration for discovery cohort (Heidelberg). **e** XY plot showing predicted TAM and TIL infiltration combined in validation cohort (UCSF/HKU, *n*=533). **f-h** Kaplan-Meier plots showing LFFR in newly-diagnosed MGMs in validation survival cohort (UCSF/HKU, *n*=221) according to (**f)** molecular group, (**g**) WHO grading, and (**h**) sex of patients. Abbreviations: HKU, University of Hongkong; LFFR, local freedom from recurrence; MGM, meningioma; ref, reference; TAM, tumor-associated macrophage; TIL, tumor-infiltrating T-lymphocyte; UCSF, University of California San Francisco. Statistical significance: *, *P*<0.05; **, *P*<0.01; ***, *P*<0.001; ****, *P*<0.0001.

**SUPPLEMENTARY TABLES**

### Supplementary Table S1: Regional variability in macrophage infiltration in different sized areas of meningioma tissue specimens

| Sample | Category | ROI | Area [mm²] | TCC | CD68+ cells  (%of TCC) |
| --- | --- | --- | --- | --- | --- |
| 1 | **TAM high** | Whole tumor  ROI2  ROI1 | 24.87  6.07  3.05 | 156,757  41,405  21,881 | 16.91%  14.86%  13.85% |
| 2 | **TAM low** | Whole tumor  ROI2  ROI1 | 24.43  6.17  3.06 | 115,476  36,853  18,040 | 1.94%  1.04%  1.38% |
| 3 | **TAM high** | Whole tumor  ROI2  ROI1 | 21.78  6.15  3.09 | 104,945  40,525  17,557 | 13.41%  13.26%  17.99% |

*Abbreviations: ROI, region of interest; TAM, tumor-associated macrophage; TCC, total cell count.*

### Supplementary Table S2: Impact of M2-like TAM infiltration on progression-free survival in the discovery cohort. Multivariate survival analysis (Cox proportional hazard model).

|  |  | n | HR | 95%-CI | *P*-value |
| --- | --- | --- | --- | --- | --- |
| M2-LIKE tam INFILTRATION | low  high | 47  47 | 1.00  2.11 | 1.11-4.01 | **0.023*** |
| Age | low  high | 47  47 | 1.00  2.06 | 1.07-3.94 | **0.030*** |
| SEX | male  female | 36  58 | 1.00  0.40 | 0.20-0.78 | **0.007**** |
| WHO grade | 1  2  3 | 27  54  13 | 1.00  1.04  1.62 | 0.48-2.27  0.60-4.35 | 0.914  0.336 |

Results of the multivariate analysis for the progression-free survival of newly-diagnosed meningioma cases calculated using Cox proportional hazard model. Abbreviations: 95%-CI, lower and upper border of 95% confidence interval; HR, hazard ratio; n, number; TAM, tumor-associated macrophage.

### **Supplementary Table S3: Correlation coefficients derived from correlation matrix of protein concentrations, TAM and TIL infiltration numbers ordered by Spearman correlation (subset of discovery cohort). Color code of cells derived from correlation matrix in Figure 2B.**


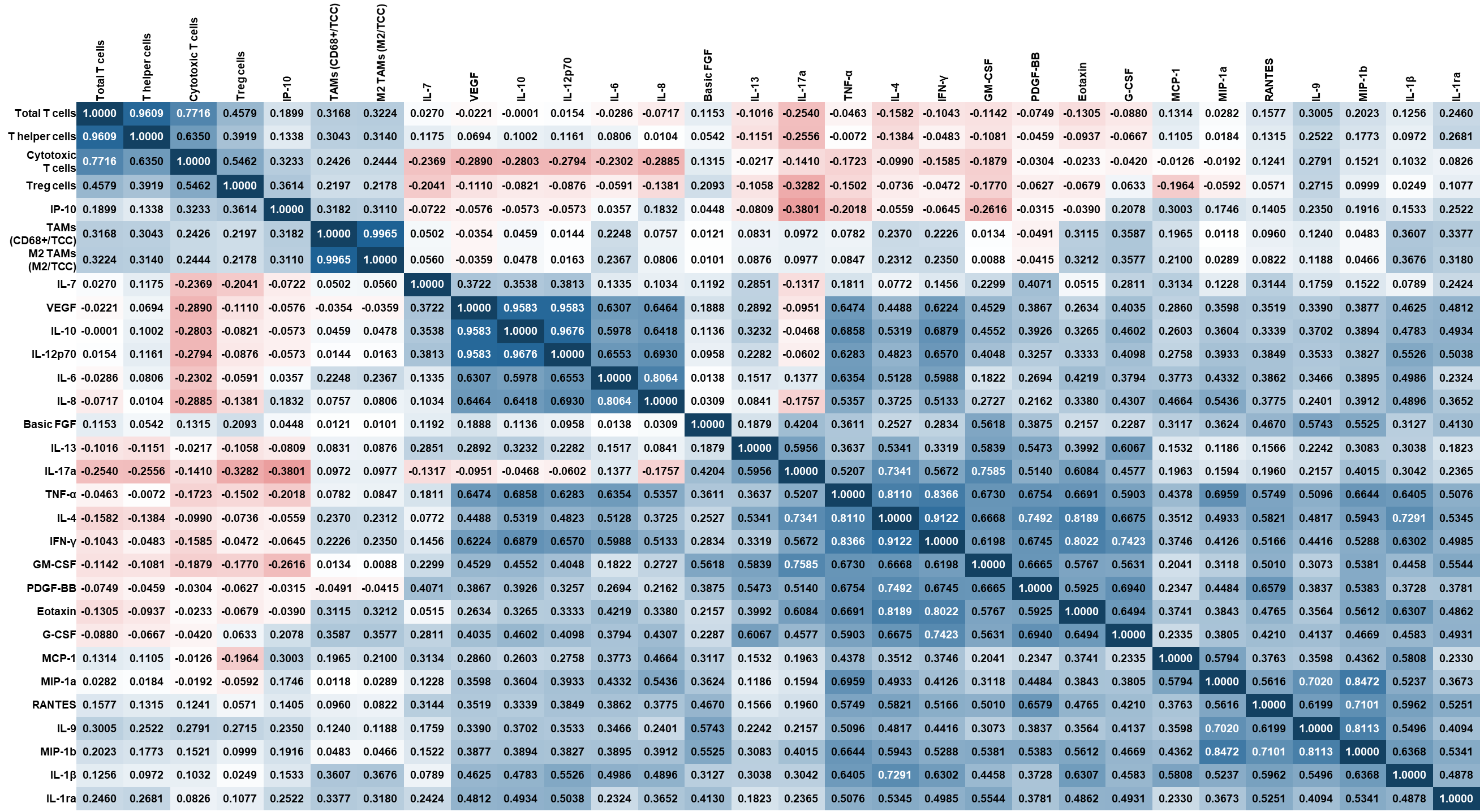


### Supplementary Table S4: *P*-values derived from correlation matrix of protein concentrations, TAM and TIL infiltration numbers ordered by Spearman correlation (subset of discovery cohort). Color code of cells derived from correlation matrix in Figure 2B.


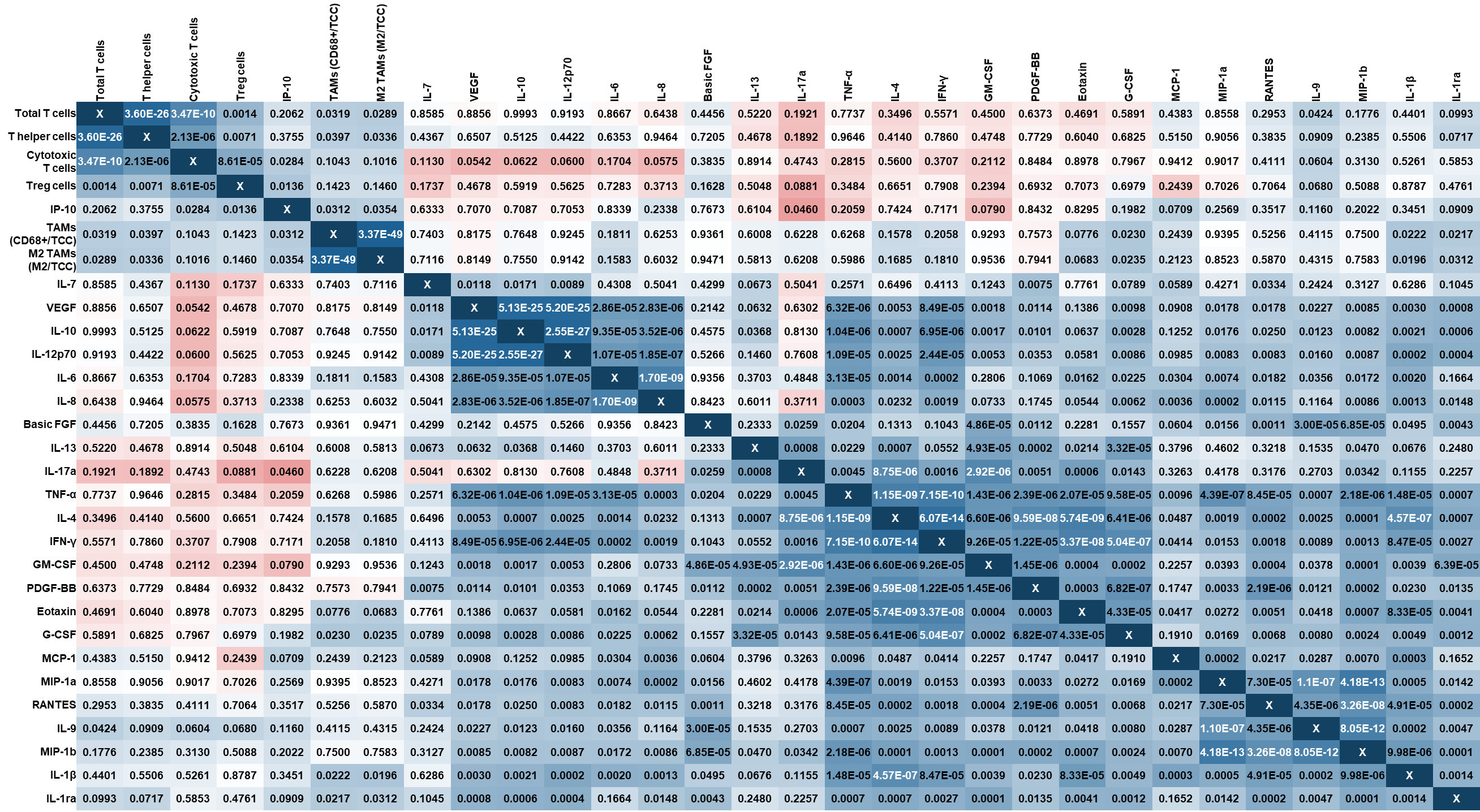


### Supplementary Table S5: Impact of TAM and TIL infiltration on progression-free survival in the discovery cohort. Multivariate survival analysis (Cox proportional hazard model).

|  |  | n | HR | 95%-CI | *P*-value |
| --- | --- | --- | --- | --- | --- |
| TAM and TIL INFILTRATION | low TAM/high TIL  low TAM/low TIL  high TAM/high TIL  high TAM/low TIL | 17  30  30  17 | 1.00  11.06  12.48  12.42 | 2.33-52.60  2.79-55.81  2.50-61.68 | **0.003****  **<0.001*****  **0.002**** |
| Age | low  high | 47  47 | 1.00  2.70 | 1.36-5.36 | **0.005**** |
| SEX | male  female | 36  58 | 1.00  0.27 | 0.13-0.57 | **<0.001***** |
| WHO grade | 1  2  3 | 27  54  13 | 1.00  1.40  1.80 | 0.64-3.08  0.64-5.01 | 0.398  0.263 |

Results of the multivariate analysis for the progression-free survival of newly-diagnosed meningioma cases calculated using Cox proportional hazard model. Abbreviations: 95%-CI, lower and upper border of 95% confidence interval; HR, hazard ratio; n, number; TAM, tumor-associated macrophage; TIL, tumor-infiltrating T-lymphocyte.

### Supplementary Table S6: Gene ontology enrichment analysis in the low TAM / high TIL group.

| **ID** | **Cluster** | **setSize** | **NES** | **p.adjust** |
| --- | --- | --- | --- | --- |
| GOBP_PEPTIDYL_TYROSINE_MODIFICATION | activation signaling | 361 | 1.7781 | 9.08E-06 |
| GOBP_POSITIVE_REGULATION_OF_MAPK_CASCADE | activation signaling | 460 | 1.8203 | 4.44E-07 |
| GOBP_REGULATION_OF_STRESS_ACTIVATED_PROTEIN_KINASE_SIGNALING_CASCADE | activation signaling | 191 | 1.9877 | 3.22E-06 |
| GOCC_EXTERNAL_SIDE_OF_PLASMA_MEMBRANE | activation signaling | 353 | 2.0867 | 4.53E-08 |
| GOBP_POSITIVE_REGULATION_OF_CYTOKINE_PRODUCTION | adaptive immune response | 450 | 1.7324 | 7.85E-06 |
| GOBP_POSITIVE_REGULATION_OF_IMMUNE_RESPONSE | adaptive immune response | 464 | 1.8074 | 4.44E-07 |
| GOBP_CELLULAR_RESPONSE_TO_PEPTIDE | adaptive immune response | 345 | 1.8509 | 3.07E-06 |
| GOBP_MONONUCLEAR_CELL_DIFFERENTIATION | adaptive immune response | 420 | 1.8549 | 3.71E-07 |
| GOBP_CELL_SUBSTRATE_ADHESION | adaptive immune response | 347 | 1.8749 | 1.24E-06 |
| GOBP_T_CELL_DIFFERENTIATION | adaptive immune response | 255 | 1.8824 | 7.85E-06 |
| GOBP_REGULATION_OF_IMMUNE_EFFECTOR_PROCESS | adaptive immune response | 329 | 1.8853 | 2.15E-06 |
| GOBP_REGULATION_OF_CELL_CELL_ADHESION | adaptive immune response | 431 | 1.9054 | 5.96E-08 |
| GOBP_POSITIVE_REGULATION_OF_CELL_CELL_ADHESION | adaptive immune response | 277 | 1.9362 | 3.85E-06 |
| GOBP_IMMUNE_RESPONSE_REGULATING_SIGNALING_PATHWAY | adaptive immune response | 376 | 1.9448 | 6.93E-08 |
| GOBP_REGULATION_OF_T_CELL_ACTIVATION | adaptive immune response | 326 | 1.9677 | 1.62E-07 |
| GOBP_REGULATION_OF_LYMPHOCYTE_ACTIVATION | adaptive immune response | 433 | 1.9688 | 4.53E-08 |
| GOBP_POSITIVE_REGULATION_OF_CELL_ADHESION | adaptive immune response | 425 | 1.9806 | 4.53E-08 |
| GOBP_CELL_ACTIVATION_INVOLVED_IN_IMMUNE_RESPONSE | adaptive immune response | 271 | 2.0028 | 4.71E-07 |
| GOBP_B_CELL_ACTIVATION | adaptive immune response | 248 | 2.0048 | 1.20E-06 |
| GOBP_T_CELL_ACTIVATION | adaptive immune response | 483 | 2.0084 | 4.53E-08 |
| GOBP_LEUKOCYTE_CELL_CELL_ADHESION | adaptive immune response | 364 | 2.0085 | 4.53E-08 |
| GOBP_LYMPHOCYTE_MEDIATED_IMMUNITY | adaptive immune response | 267 | 2.0227 | 3.07E-07 |
| GOBP_ADAPTIVE_IMMUNE_RESPONSE | adaptive immune response | 416 | 2.0384 | 4.53E-08 |
| GOBP_ADAPTIVE_IMMUNE_RESPONSE_BASED_ON_SOMATIC_RECOMBINATION_OF_IMMUNE_RECEPTORS_BUILT_FROM_IMMUNOGLOBULIN_SUPERFAMILY_DOMAINS | adaptive immune response | 275 | 2.0523 | 2.46E-07 |
| GOBP_POSITIVE_REGULATION_OF_CELL_ACTIVATION | adaptive immune response | 334 | 2.0842 | 4.53E-08 |
| GOBP_LEUKOCYTE_MEDIATED_IMMUNITY | adaptive immune response | 352 | 2.0945 | 4.53E-08 |
| GOBP_POSITIVE_REGULATION_OF_LEUKOCYTE_CELL_CELL_ADHESION | adaptive immune response | 236 | 2.1406 | 4.53E-08 |
| GOBP_LEUKOCYTE_PROLIFERATION | adaptive immune response | 322 | 2.1507 | 4.53E-08 |
| GOBP_POSITIVE_REGULATION_OF_T_CELL_PROLIFERATION | adaptive immune response | 102 | 2.1710 | 3.38E-06 |
| GOBP_REGULATION_OF_LEUKOCYTE_PROLIFERATION | adaptive immune response | 247 | 2.1727 | 4.53E-08 |
| GOBP_B_CELL_MEDIATED_IMMUNITY | adaptive immune response | 130 | 2.1805 | 6.56E-07 |
| GOMF_IMMUNE_RECEPTOR_ACTIVITY | adaptive immune response | 130 | 2.2758 | 6.03E-08 |
| GOBP_POSITIVE_REGULATION_OF_LEUKOCYTE_PROLIFERATION | adaptive immune response | 156 | 2.3267 | 4.53E-08 |
| HP_ABNORMAL_CORTICAL_GYRATION | cancer induced abnormality | 376 | -1.8874 | 2.92E-07 |
| HP_ABNORMALITY_OF_NEURONAL_MIGRATION | cancer induced abnormality | 452 | -1.8038 | 2.83E-07 |
| HP_ABNORMAL_MYELINATION | cancer induced abnormality | 482 | -1.6860 | 4.67E-06 |
| GOBP_CELL_ADHESION_MEDIATED_BY_INTEGRIN | cancer induced abnormality | 81 | 2.3882 | 6.93E-08 |
| GOBP_CYTOKINE_MEDIATED_SIGNALING_PATHWAY | cytokine signaling | 452 | 1.7546 | 1.91E-06 |
| GOCC_RECEPTOR_COMPLEX | cytokine signaling | 397 | 1.7726 | 8.38E-06 |
| GOBP_WOUND_HEALING | cytokine signaling | 408 | 1.8225 | 1.64E-06 |
| GOMF_CYTOKINE_BINDING | cytokine signaling | 129 | 2.3332 | 4.53E-08 |
| GOBP_REGULATION_OF_CHEMOTAXIS | leucocyte chemotaxis | 211 | 2.0372 | 5.57E-07 |
| GOBP_POSITIVE_REGULATION_OF_LEUKOCYTE_MIGRATION | leucocyte chemotaxis | 132 | 2.1053 | 3.40E-06 |
| GOBP_REGULATION_OF_LEUKOCYTE_CHEMOTAXIS | leucocyte chemotaxis | 112 | 2.1423 | 7.48E-06 |
| GOBP_MONONUCLEAR_CELL_MIGRATION | leucocyte chemotaxis | 181 | 2.1607 | 5.78E-08 |
| GOBP_POSITIVE_REGULATION_OF_CHEMOTAXIS | leucocyte chemotaxis | 133 | 2.1627 | 9.32E-07 |
| GOBP_REGULATION_OF_LEUKOCYTE_MIGRATION | leucocyte chemotaxis | 202 | 2.1755 | 6.93E-08 |
| GOBP_LEUKOCYTE_MIGRATION | leucocyte chemotaxis | 357 | 2.1857 | 4.53E-08 |
| GOBP_CELL_CHEMOTAXIS | leucocyte chemotaxis | 283 | 2.2076 | 4.53E-08 |
| GOBP_LEUKOCYTE_CHEMOTAXIS | leucocyte chemotaxis | 213 | 2.2448 | 4.53E-08 |
| GOBP_MYELOID_LEUKOCYTE_MIGRATION | leucocyte chemotaxis | 209 | 2.2718 | 4.53E-08 |
| GOBP_MACROPHAGE_CHEMOTAXIS | leucocyte chemotaxis | 36 | 2.3748 | 8.53E-06 |
| GOBP_REGULATION_OF_MACROPHAGE_MIGRATION | leucocyte chemotaxis | 40 | 2.4002 | 1.97E-06 |
| GOBP_MACROPHAGE_MIGRATION | leucocyte chemotaxis | 53 | 2.4913 | 6.93E-08 |
| GOBP_PEPTIDE_ANTIGEN_ASSEMBLY_WITH_MHC_CLASS_II_PROTEIN_COMPLEX | MHC-class-II presentation | 13 | 2.3293 | 2.52E-06 |
| GOBP_ANTIGEN_PROCESSING_AND_PRESENTATION_OF_EXOGENOUS_PEPTIDE_ANTIGEN_VIA_MHC_CLASS_II | MHC-class-II presentation | 25 | 2.3985 | 9.93E-06 |
| GOCC_MHC_CLASS_II_PROTEIN_COMPLEX | MHC-class-II presentation | 14 | 2.4040 | 1.45E-06 |
| GOCC_MHC_PROTEIN_COMPLEX | MHC-class-II presentation | 21 | 2.4443 | 3.07E-06 |
| GOBP_ANTIGEN_PROCESSING_AND_PRESENTATION_OF_PEPTIDE_OR_POLYSACCHARIDE_ANTIGEN_VIA_MHC_CLASS_II | MHC-class-II presentation | 29 | 2.4490 | 3.85E-06 |
| GOMF_MHC_CLASS_II_PROTEIN_COMPLEX_BINDING | MHC-class-II presentation | 23 | 2.4505 | 2.54E-06 |
| GOBP_NCRNA_PROCESSING | nucleolar/rRNA processing | 330 | -2.0421 | 4.53E-08 |
| GOBP_RIBOSOME_BIOGENESIS | nucleolar/rRNA processing | 268 | -1.9697 | 1.25E-06 |
| GOBP_NCRNA_METABOLIC_PROCESS | nucleolar/rRNA processing | 440 | -1.8517 | 1.50E-07 |
| GOBP_RIBONUCLEOPROTEIN_COMPLEX_BIOGENESIS | nucleolar/rRNA processing | 377 | -1.8087 | 3.22E-06 |
| GOBP_MICROTUBULE_BASED_TRANSPORT | tumor cell proliferation | 165 | -2.0064 | 7.62E-06 |
| GOBP_CHROMOSOME_SEGREGATION | tumor cell proliferation | 304 | -1.9382 | 4.58E-07 |
| GOBP_MICROTUBULE_BASED_MOVEMENT | tumor cell proliferation | 315 | -1.9014 | 1.91E-06 |
| GOMF_TUBULIN_BINDING | tumor cell proliferation | 325 | -1.8437 | 9.93E-06 |
| GOCC_MICROTUBULE | tumor cell proliferation | 392 | -1.8286 | 9.56E-07 |
| GOCC_SPINDLE | tumor cell proliferation | 357 | -1.8069 | 7.62E-06 |

### Supplementary Table S7: Clinicopathological characteristics of patients with newly-diagnosed and recurrent meningiomas of the validation cohort.

|  | **Newly-diagnosed MGMs (*n*=456)** | | |  | **Recurrent MGMs (*n*=109)** | | |
| --- | --- | --- | --- | --- | --- | --- | --- |
| **Variable** | ***n*** | **Patients (%)** | **Median (range)** |  | ***n*** | **Patients (%)** | **Median (range)** |
| Sex | | | | | | | |
| Male | 147 | 32.24 |  |  | 46 | 42.20 |  |
| Female | 309 | 67.76 |  |  | 63 | 57.80 |  |
| Age at time of surgery (years) | | | 57.5 (11.0-90.6) |  |  |  | 57.1 (20.7-87.5) |
| WHO grade |  |  |  |  |  |  |  |
| WHO grade 1 | 348 | 76.32 |  |  | 40 | 36.70 |  |
| WHO grade 2 | 95 | 20.83 |  |  | 47 | 43.12 |  |
| WHO grade 3 | 13 | 2.85 |  |  | 22 | 20.18 |  |
| Resection grade | | | | | | | |
| GTR | 335 | 73.46 |  |  | 59 | 54.13 |  |
| STR | 115 | 25.22 |  |  | 43 | 39.45 |  |
| NA | 6 | 1.32 |  |  | 7 | 6.42 |  |
| Molecular group | | | | | | | |
| Merlin-intact | 132 | 28.95 |  |  | 8 | 7.34 |  |
| Immune-enriched | 171 | 37.50 |  |  | 35 | 32.11 |  |
| Hypermitotic | 87 | 19.08 |  |  | 52 | 47.71 |  |
| NA | 66 | 14.47 |  |  | 14 | 12.84 |  |
| Abbreviations: GTR, gross total resection; STR, subtotal resection; n, number; NA, not available; MGM, meningioma | | | | | | | |

### Supplementary Table S8: CpG sites for TAMs and TILs as DNA methylation-based signatures for predicting immune cell infiltration.

|  | **CpGs for TILs (n=63)** | | **CpGs for TAMs (n=43)** | |
| --- | --- | --- | --- | --- |
| **#** | **Coefficient names** | **Coefficient value** | **Coefficient names** | **Coefficient value** |
|  | Intercept | 50.99662 | Intercept | 250.6186 |
| 1 | cg18627816 | -0.80422 | cg14028684 | -1.52753 |
| 2 | cg19863740 | -0.30395 | cg16273546 | -4.86964 |
| 3 | cg05540649 | -3.65017 | cg18735114 | -11.125 |
| 4 | cg02003183 | -0.08023 | cg11343938 | -2.62638 |
| 5 | cg14094409 | -1.72898 | cg05579025 | -6.87242 |
| 6 | cg12004641 | -0.59057 | cg10694914 | -1.36755 |
| 7 | cg04835359 | -4.63 | cg24268698 | -8.09043 |
| 8 | cg12451280 | -3.16943 | cg26420795 | -0.66689 |
| 9 | cg02423817 | -0.34577 | cg04372533 | -5.21851 |
| 10 | cg18520034 | -1.91984 | cg17168875 | -3.60735 |
| 11 | cg15328005 | -1.74016 | cg25316856 | -9.18789 |
| 12 | cg04655995 | -0.20871 | cg00826610 | -41.4131 |
| 13 | cg02710553 | -0.53399 | cg11772801 | -0.48005 |
| 14 | cg20417024 | -0.02301 | cg20133046 | -3.89875 |
| 15 | cg01888251 | -0.29079 | cg01484266 | -4.8215 |
| 16 | cg02902617 | -0.21999 | cg01336233 | -16.1101 |
| 17 | cg08636224 | -1.04113 | cg20125028 | -27.3284 |
| 18 | cg02690148 | -1.43192 | cg11231069 | -1.54133 |
| 19 | cg14983777 | -0.55447 | cg12899381 | -1.50478 |
| 20 | cg09032544 | -0.66038 | cg14622996 | -0.69262 |
| 21 | cg03421440 | -0.74258 | cg18211585 | -6.26384 |
| 22 | cg15885274 | -0.91767 | cg06217314 | -0.15211 |
| 23 | cg23590302 | -3.9209 | cg14544289 | -0.687 |
| 24 | cg17156558 | -1.83274 | cg02902617 | -0.04041 |
| 25 | cg24142603 | -0.38726 | cg16882301 | -9.46649 |
| 26 | cg21810379 | -0.96147 | cg10677351 | -14.6669 |
| 27 | cg12818481 | -2.19247 | cg01281061 | -7.08341 |
| 28 | cg00242950 | -0.26383 | cg25049486 | -16.819 |
| 29 | cg01757548 | -0.39938 | cg26305504 | -0.07334 |
| 30 | cg11152463 | -0.2928 | cg18129961 | -6.61907 |
| 31 | cg10559742 | -0.01645 | cg04418529 | -35.0905 |
| 32 | cg10591385 | -0.4243 | cg20631997 | -1.34594 |
| 33 | cg16363586 | -0.17025 | cg21274607 | -8.10168 |
| 34 | cg09779405 | -0.63096 | cg16957824 | -0.44488 |
| 35 | cg04131405 | -0.09641 | cg01431057 | -3.38474 |
| 36 | cg17136073 | -0.18379 | cg05866737 | -1.84502 |
| 37 | cg02957290 | -1.5256 | cg14789529 | -1.98055 |
| 38 | cg23575668 | -0.25278 | cg26610167 | -0.59712 |
| 39 | cg10193870 | -0.838 | cg21555346 | -0.66736 |
| 40 | cg02811067 | -0.19277 | cg23878206 | -0.21088 |
| 41 | cg11697120 | -0.13665 | cg12051260 | -17.9253 |
| 42 | cg08400494 | -0.06777 | cg25824218 | -0.40864 |
| 43 | cg04029027 | -0.20445 | cg14864369 | -14.0739 |
| 44 | cg22842189 | -0.04658 |  |  |
| 45 | cg01047779 | -0.05358 |  |  |
| 46 | cg03000848 | -0.56409 |  |  |
| 47 | cg05044173 | -0.21744 |  |  |
| 48 | cg11606444 | -0.00506 |  |  |
| 49 | cg15709435 | -1.64962 |  |  |
| 50 | cg19627093 | -0.50222 |  |  |
| 51 | cg07444005 | -0.7783 |  |  |
| 52 | cg20496896 | -0.29121 |  |  |
| 53 | cg14205576 | -1.06428 |  |  |
| 54 | cg11236515 | -1.78062 |  |  |
| 55 | cg04820362 | -1.51774 |  |  |
| 56 | cg01180835 | -0.86239 |  |  |
| 57 | cg05059108 | -0.36898 |  |  |
| 58 | cg17954852 | -0.58133 |  |  |
| 59 | cg24411075 | -0.1151 |  |  |
| 60 | cg24862189 | -0.13045 |  |  |
| 61 | cg10200001 | -0.54373 |  |  |
| 62 | cg02537014 | -1.42129 |  |  |
| 63 | cg18501409 | -0.32535 |  |  |
| Abbreviations: TAM, tumor-associated macrophage; TIL, tumor-infiltrating T-lymphocyte. | | | | |

### Supplementary Table S9: Clinicopathological characteristics of patients with newly-diagnosed meningiomas of the validation cohort for survival analysis.

|  | **Newly-diagnosed MGMs (*n*=221)** | | |  |
| --- | --- | --- | --- | --- |
| **Variable** | ***n*** | **Patients (%)** | **Median (range)** |  |
| Sex |  |  |  |  |
| Male | 76 | 34.39 |  |  |
| Female | 145 | 65.61 |  |  |
| Age at time of surgery (years) | | | 57.1 (11.0-90.6) |  |
| WHO grade |  |  |  |  |
| WHO grade 1 | 178 | 80.54 |  |  |
| WHO grade 2 | 40 | 18.10 |  |  |
| WHO grade 3 | 3 | 1.36 |  |  |
| Resection grade |  |  |  |  |
| GTR | 221 | 100.00 |  |  |
| STR | 0 | 0.00 |  |  |
| NA | 0 | 0.00 |  |  |
| Molecular group |  |  |  |  |
| Merlin-intact | 78 | 35.29 |  |  |
| Immune-enriched | 95 | 42.99 |  |  |
| Hypermitotic | 48 | 21.72 |  |  |
| NA | 0 |  |  |  |
| Abbreviations: GTR, gross total resection; STR, subtotal resection; n, number; NA, not available; MGM, meningioma | | | | |

### Supplementary Table S10: Impact of TIL infiltration on recurrence-free survival in the validation cohort. Multivariate survival analysis (Cox proportional hazard model).

|  |  | n | HR | 95%-CI | *P*-value |
| --- | --- | --- | --- | --- | --- |
| TIL INFILTRATION | low  high | 111  110 | 1.00  2.11 | 0.29-0.91 | **0.022*** |
| MOLECULAR GROUP | hypermitotic  immune-enriched  merlin-intact | 48  95  78 | 1.00  0.80  0.36 | 0.42-1.52  0.18-0.74 | 0.493  **0.006**** |
| WHO grade | 1  2  3 | 178  40  3 | 1.00  1.93  10.91 | 1.04-3.57  3.18-37.44 | **0.036***  ***P* < 0.001***** |
| SEX | male  female | 76  145 | 1.00  0.74 | 0.43-1.28 | 0.283 |

Results of the multivariate analysis for the recurrence-free survival of newly-diagnosed meningioma cases calculated using Cox proportional hazard model. Abbreviations: 95%-CI, lower and upper border of 95% confidence interval; HR, hazard ratio; n, number; TIL, tumor-infiltrating T-lymphocyte.

### Supplementary Table S11: Impact of TAM infiltration on recurrence-free survival in the validation cohort. Multivariate survival analysis (Cox proportional hazard model).

|  |  | n | HR | 95%-CI | *P*-value |
| --- | --- | --- | --- | --- | --- |
| TAM INFILTRATION | low  high | 111  110 | 1.00  2.22 | 1.19-4.14 | **0.01**** |
| MOLECULAR GROUP | hypermitotic  immune-enriched  merlin-intact | 48  95  78 | 1.00  0.44  0.42 | 0.23-0.86  0.21-0.86 | **0.02***  **0.02*** |
| WHO grade | 1  2  3 | 178  40  3 | 1.00  1.78  12.47 | 0.97-3.24  3.64-42.76 | **0.06**  ***P* < 0.001***** |
| SEX | male  female | 76  145 | 1.00  0.80 | 0.47-1.36 | 0.410 |

Results of the multivariate analysis for the recurrence-free survival of newly-diagnosed meningioma cases calculated using Cox proportional hazard model. Abbreviations: 95%-CI, lower and upper border of 95% confidence interval; HR, hazard ratio; n, number; TAM, tumor-associated macrophage.

### Supplementary Table S12: Impact of TAM and TIL infiltration on recurrence-free survival in the validation cohort. Multivariate survival analysis (Cox proportional hazard model).

|  |  | n | HR | 95%-CI | *P*-value |
| --- | --- | --- | --- | --- | --- |
| TAM and TIL INFILTRATION | low TAM/high TIL  low TAM/low TIL  high TAM/high TIL  high TAM/low TIL | 32  79  78  32 | 1.00  8.38  9.69  22.80 | 1.12-62.60  1.26-74.30  2.96-175.46 | **0.038***  **0.029***  **0.003**** |
| MOLECULAR GROUP | hypermitotic  immune-enriched  merlin-intact | 48  95  78 | 1.00  0.51  0.40 | 0.26-0.99  0.20-0.83 | **0.048***  **0.013*** |
| WHO grade | 1  2  3 | 178  40  3 | 1.00  2.02  12.62 | 1.10-3.73  3.61-44.09 | **0.024***  ***P* < 0.001***** |
| SEX | male  female | 76  145 | 1.00  0.74 | 0.43-1.27 | 0.280 |

Results of the multivariate analysis for the recurrence-free survival of newly-diagnosed meningioma cases calculated using Cox proportional hazard model. Abbreviations: 95%-CI, lower and upper border of 95% confidence interval; HR, hazard ratio; n, number; TAM, tumor-associated macrophage; TIL, tumor-infiltrating T-lymphocyte.
